# Variability in Arterial Stiffness and Vascular Endothelial Function After COVID-19 During 1.5 Years of Follow-Up—Systematic Review and Meta-Analysis

**DOI:** 10.3390/life15040520

**Published:** 2025-03-21

**Authors:** Danuta Loboda, Krzysztof S. Golba, Piotr Gurowiec, Aelita Bredelytė, Artūras Razbadauskas, Beata Sarecka-Hujar

**Affiliations:** 1 Department of Electrocardiology and Heart Failure, Medical University of Silesia in Katowice, 40-635 Katowice, Poland; kgolba@sum.edu.pl (K.S.G.); piotr.gurowiec@sum.edu.pl (P.G.); 2 Faculty of Health Sciences, Klaipėda University, LT-92294 Klaipeda, Lithuania; aelita.bredelyte@ku.lt (A.B.); arturas.razbadauskas@ku.lt (A.R.); 3 Chemotherapy Unit, Department of Oncology, Klaipeda University Hospital, LT-92288 Klaipeda, Lithuania; 4 Department of Basic Biomedical Science, Faculty of Pharmaceutical Sciences in Sosnowiec, Medical University of Silesia in Katowice, 41-200 Sosnowiec, Poland; bsarecka-hujar@sum.edu.pl

**Keywords:** arterial stiffness, atherosclerotic cardiovascular disease, brachial flow-mediated dilation, carotid-femoral pulse wave velocity, endothelium-dependent vasodilation, long COVID-19, post-COVID-19, SARS-CoV-2, vascular aging

## Abstract

Increasing long-term observations suggest that coronavirus disease 2019 (COVID-19) vasculopathy may persist even 1.5 years after the acute phase, potentially accelerating the development of atherosclerotic cardiovascular diseases. This study systematically reviewed the variability of brachial flow-mediated dilation (FMD) and carotid-femoral pulse wave velocity (cfPWV) from the acute phase of COVID-19 through 16 months of follow-up (F/U). Databases including PubMed, Web of Science, MEDLINE, and Embase were screened for a meta-analysis without language or date restrictions (PROSPERO reference CRD42025642888, last search conducted on 1 February 2025). The quality of the included studies was assessed using the Newcastle–Ottawa Quality Scale. We considered all studies (interventional pre-post studies, prospective observational studies, prospective randomized, and non-randomized trials) that assessed FMD or cfPWV in adults (aged ≥ 18 years) with or after laboratory-confirmed COVID-19 compared with non-COVID-19 controls or that assessed changes in these parameters during the F/U. Twenty-one studies reported differences in FMD, and 18 studies examined cfPWV between COVID-19 patients and control groups during various stages: acute/subacute COVID-19 (≤30 days from disease onset), early (>30–90 days), mid-term (>90–180 days), late (>180–270 days), and very late (>270 days) post-COVID-19 recovery. Six studies assessed variability in FMD, while nine did so for cfPWV during the F/U. Data from 14 FMD studies (627 cases and 694 controls) and 15 cfPWV studies (578 cases and 703 controls) were included in our meta-analysis. FMD showed a significant decrease compared to controls during the acute/subacute phase (standardized mean difference [SMD]= −2.02, *p* < 0.001), with partial improvements noted from the acute/subacute phase to early recovery (SMD = 0.95, *p* < 0.001) and from early to mid-term recovery (SMD = 0.92, *p* = 0.006). Normalization compared to controls was observed in late recovery (SMD = 0.12, *p* = 0.69). In contrast, cfPWV values, which were higher than controls in the acute/subacute phase (SMD = 1.27, *p* < 0.001), remained elevated throughout the F/U, with no significant changes except for a decrease from mid-term to very late recovery (SMD= −0.39, *p* < 0.001). In the very late recovery, cfPWV values remained higher than those of controls (SMD = 0.45, *p* = 0.010). In the manuscript, we discuss how various factors, including the severity of acute COVID-19, the persistence of long-term COVID-19 syndrome, and the patient’s initial vascular age, depending on metrics age and cardiovascular risk factors, influenced the time and degree of FMD and cfPWV improvement.

## 1. Introduction

Severe acute respiratory syndrome coronavirus 2 (SARS-CoV-2) leads to hyperinflammation, hypercoagulability, and the dysregulation of the immune system, resulting in damage to the vascular endothelium, lung injury, thromboembolic events, and complications affecting the cardiovascular (CV) and cerebrovascular systems, as well as nephropathy and retinopathy [1,2,3]. The initial unfavorable changes are initiated when SARS-CoV-2 binds to the angiotensin-converting enzyme 2 (ACE2) receptor, causing its internalization and downregulation. This process reduces ACE2’s catalytic activity towards angiotensin II. Consequently, elevated levels of angiotensin II cause microvascular vasoconstriction and vascular inflammation by decreasing the bioavailability of nitric oxide (NO) and increasing the endothelial production of reactive oxygen species, matrix metalloproteases, inflammatory cytokines, and adhesion molecules [4,5,6,7]. Furthermore, the interaction between SARS-CoV-2 spike proteins—found during the acute phase and persisting in organs during the post-COVID-19 period—and ACE2 receptors, along with the accumulation of angiotensin II, can lead to acute increases in blood pressure and heighten the risk of new-onset hypertension [8,9].

Histopathological examinations, multimodal imaging, capillaroscopy, and optical coherence tomography angiography (OCTA) have all revealed various macro- and microvascular alterations, indicating a heightened inflammatory burden in the vessels of individuals who have had or are currently experiencing coronavirus disease 2019 (COVID-19) [5,7,10,11,12,13,14,15,16,17,18,19,20,21,22]. Post-mortem revealed the presence of viral bodies within endothelial cells, an accumulation of inflammatory cells, endothelial and inflammatory cell apoptosis [5], and diffuse arteriole thrombi [17] across vascular beds of various organs in acute COVID-19 fatalities. In vitro and in vivo analysis, damage to endothelial barriers with increased endothelial permeability was documented [7,18]. During the acute phase of COVID-19, OCTA showed a COVID-related decrease in central retinal vessel density, which was associated with disease severity [19]. Notably, the retinal vascular changes were not reversible within 12 months after recovery. These changes were linked to the severity of inflammation (as indicated by the level of C-reactive protein) and renal dysfunction during the acute phase and were influenced by an aortic stiffness measured during follow-up (F/U) [13]. Moreover, in a nail fold video capillaroscopy, capillary changes, such as capillary ramifications, loss of capillaries, and caliber variability, with an overall higher microangiopathy evolution score were more frequently observed in the post-COVID-19 late recovery period than in patients with ASCVD and healthy controls [10]. Additionally, fibrin microthrombi within cardiac arterioles [20] increased the perfused boundary region, indicating reduced endothelial glycocalyx shedding, and an impaired coronary flow reserve compared to healthy controls persisting up to 4–12 months of recovery [11,12] was documented. Residual cardiovascular alterations at 4 months were proportional to markers of oxidative stress and endothelial dysfunction [11]. In larger peripheral arteries (the common carotid, axillary, or superficial femoral artery), the intima-media thickness was increased compared to healthy controls [10,15,21] but comparable to patients with atherosclerotic CV disease (ASCVD) [10]. Some authors observed features of large vessel vasculitis in positron emission tomography/computed tomography with 2-deoxy-2-[18F]-fluoro-D-glucose in patients with persisting post-COVID-19 symptoms [22].

It has been proven that post-COVID-19 inflammation persists over a year after the initiating stimulus, i.e., SARS-CoV-2 infection, has subsided, even in previously healthy individuals [10,23,24,25]. It can be assumed that the resulting endothelial dysfunction with diminished NO bioavailability [4,10,11,26,27,28,29,30,31,32] and arterial stiffness (AS) [10,11,21,33,34,35,36,37,38,39] play a role in the pathogenesis of atherosclerosis [6], accelerate vascular aging [40], increase the risk of new ASCVD [3,41,42,43,44], and predict CV outcome in COVID-19 convalescents [45,46,47]. The AS in post-COVID-19 convalescents was significantly related to ASCVD risk when assessed using the Systemic Coronary Risk Evaluation 2 algorithm and better discriminated the low to moderate and the very high ASCVD risk groups than optimal blood pressure, correct body mass index (BMI), and normal lipid parameters, which are considered in risk estimation in everyday practice [48].

Currently, both endothelial function and AS can be measured noninvasively. One method for assessing endothelium-dependent vasodilation is brachial flow-mediated dilation (FMD) [49,50]. In this procedure, a pneumatic cuff is placed on the forearm and inflated to a pressure that temporarily stops blood flow in the artery. Once the cuff is deflated, the blood flow increases shear stress on the arterial wall, which stimulates the production of NO and leads to vasodilation. FMD is measured using ultrasound, and the results are calculated as the percentage change between the average brachial artery diameter after reactive hyperemia and the baseline diameter, divided by the baseline diameter [50]. Systemic inflammation and major CV risk factors are independent predictors of endothelial dysfunction as assessed using FMD [51,52,53]. Therefore, FMD serves as a marker for both subclinical and advanced arteriosclerosis [49,50]. Research has shown that each 1% reduction in FMD is associated with a 9–13% increased risk of CV events, such as acute coronary syndrome, stroke, or death, in the general population [54,55].

Carotid-femoral pulse wave velocity (cfPWV) is considered the measure of reference of central AS [56,57]. The evaluation of cfPWV involves measuring the pulse transit time along the large arteries that connect the heart and the peripheral resistance vessels. Several factors, including the elasticity and thickness of the arterial walls, influence the cfPWV value [57]. The most commonly used devices simultaneously record the arterial pulse waves in the carotid and femoral arteries by employing mechanotransduction probes, which synchronize these signals with the R wave of the electrocardiogram [57]. The severity of AS is primarily determined by age, blood pressure, and various classical and non-classical CV risk factors, including chronic inflammation [53,58,59]. Consequently, it is considered a measure of vascular aging, reflecting the biological age of the arteries rather than their chronological age [60]. Early vascular aging is a predictor of adverse CV outcomes [61,62,63], with a 1 m/s increase in cfPWV associated with a 14–15% higher risk of CV events, CV mortality, and all-cause mortality in the general population [61].

Long-term follow-up studies of large cohorts may provide valuable insights into the duration and potential reversibility of post-COVID-19 vasculopathy, contributing factors, and prognostic implications. This study aimed to systematically review the variability of FMD and cfPWV from the acute and subacute COVID-19 phase through early, mid-term, late, and very late recovery, up to a year and a half of F/U.

## 2. Materials and Methods

### 2.1. Search Strategy and Inclusion/Exclusion Criteria

This study is a systematic review and meta-analysis conducted according to the Preferred Reporting Items for Systematic Reviews and Meta-Analyses (PRISMA) standards [64] shown in Supplementary Table S1.

### 2.2. Search Strategy and Inclusion/Exclusion Criteria

We specify the target population by the “population”, “intervention”, “comparison”, and “outcome” (PICO) model to answer the following research question: what is the variability of vascular endothelial function (assessed as FMD) and arterial stiffness (assessed as cfPWV) in patients recovered from COVID-19 over time, and how long can vasculopathy persist after the acute phase of the disease compared to non-COVID-19 controls?

According to a predefined protocol (see: International Prospective Registry of Systematic Reviews “PROSPERO”, ref. CRD42025642888), two independent investigators (D.L. and B.S.-H.) searched four databases (PubMed, Web of Science, MEDLINE, Embase) to identify available data published before 1 February 2025, with the use of the following keywords: (1) (“flow mediated dilation” or “endothelium dependent dilation” or “nitrate mediated dilation”) and (“covid” or “post covid” or “long covid” or “sars cov 2”), and (2) (“carotid femoral pulse wave velocity” or “arterial stiffness”) and (“covid” or “post covid” or “long covid” or “sars cov 2”). At the database search stage, no filters, language restrictions, or automation tools were used to avoid unintentionally removing publications. However, in the next step, we excluded case series without a control group, case reports, abstracts from scientific conferences, unpublished research, reviews, and ex vivo or animal studies. We did not include studies on children. In addition, we reviewed the reference lists of relevant publications for manuscripts that could be included.

Eventually, we included all studies (interventional pre-post studies, prospective observational studies (cohort, cross-sectional, case-control), prospective randomized and non-randomized trials) that assessed FMD or cfPWV in adults (aged 18 years or older) with or after laboratory-confirmed COVID-19 compared with non-COVID-19 controls or that assessed changes in these parameters during the F/U. We excluded those studies that did not specify the time from the onset of COVID-19 symptoms/SARS-CoV-2 real-time-polymerase chain reaction test/hospital discharge to the time of study inclusion and measurements.

### 2.3. Data Extraction and Quality Assessment

From each study, independent researchers (D.L., B.S-H., P.G., A.R., and A.B.) extracted and rechecked the following data: study characteristics (first author, publication year, country of origin, study design and methodology), cohort characteristics (sample size (cases and controls), anthropometric data, clinical data (regarding the severity of acute COVID-19, persistent symptoms during convalescence/presence of long-term COVID-19 syndrome), and CV risk factors/ASCVD), and FMD (%) or cfPWV (m/s) values. Two authors (D.L. and B.S.-H.) used the Newcastle–Ottawa Scale (NOS) to assess the methodological quality of the included studies. In case of discrepancies in the quality assessment, any doubts were resolved by discussion between the reviewing authors and agreement on a common assessment. A maximum score of nine points can be set on the NOS scale for each study. Studies scoring less than six points were considered a high bias risk [65].

We summarized significant findings regarding changes in FMD and cfPWV in the predefined phase of disease and convalescence, namely, in the acute/subacute COVID-19 (≤30 days from disease onset) and early (>30–90 days), mid-term (>90–180 days), late (>180–270 days), and very late (>270 days) post-COVID-19 recovery.

### 2.4. Statistical Analysis and Assessment of Bias

Statistical analyses were performed using the Review Manager software (RevMan version 5.4 Cochrane, London, UK) and MedCalc software (version 23.1.3.; MedCalc Software Ltd., Ostend, Belgium). We included only FMD or cfPWV data for the final meta-analysis, which were reported as mean values with standard deviation.

A measure of heterogeneity in the I^2^ test was obtained for each comparison. The results of the I^2^ test express the proportion of dispersion due to heterogeneity, i.e., I^2^ at 25%, 50%, and 75% suggest low, intermediate, and high inconsistency, respectively. For each comparison of FMD and cfPWV between COVID-19 patients and controls, as well as between COVID-19 patients during the F/U (e.g., acute period vs. early recovery period), the standardized mean difference (SMD) with a 95% confidence interval (CI) was calculated in the meta-analysis. SMD allows us to compare the results between two groups by placing them on a standard deviation scale. Pooled analyses were performed using random effects models (REMs), which assume that the proper effect size varies across studies; therefore, the studies in a given analysis are a random sample of the effect sizes that could have been observed. In a random effects analysis, a very small or extensive study cannot be discounted by giving them very little or considerable weight, respectively, because the estimate provided by that study may be imprecise. However, it is information about an effect no other study has estimated. In this model, the average effect across a range of studies is estimated, and none of them do not overstate the overall estimate. The random effects model accounts for unexplained heterogeneity and is often used when the number of studies included in the meta-analysis is small.

The publication bias was evaluated visually by inspecting funnel plots and performing Egger’s and Begg’s tests. In addition, to evaluate the stability of the results, sensitivity analyses were made by sequentially excluding each study.

## 3. Results

### 3.1. Study Characteristics

After eliminating duplicates and nonsignificant results, we considered 60 publications for analysis. We excluded 27 records because of inappropriate publication type or study design (e.g., lack of a non-COVID-19 control group) [21,32,34,66,67,68,69,70,71,72,73,74,75,76,77,78], inappropriate study cohort (e.g., children/adolescents, very narrow population with specific risk factors, or subjected to intervention) [13,79,80,81,82,83,84,85], or unspecified time/too-wide time range from COVID-19 onset to study enrollment [86,87,88]. All considered studies were of high quality on the NOS scale. Supplementary Figure S1 shows a PRISMA [64] systematic review flow diagram.

As a result, we included 21 studies that reported FMD, and 18 that reported cfPWV differences between laboratory-confirmed COVID-19 patients and non-COVID-19 control groups in the predefined acute/subacute (eight studies) [28,33,89,90,91,92,93,94], early recovery (11 studies) [26,28,91,95,96,97,98,99,100,101,102], mid-term recovery (nine studies) [11,27,35,91,102,103,104,105,106], late recovery (three studies) [10,28,99], and very late recovery (five studies) [12,29,35,107,108] post-COVID-19 periods. A total of 17 studies compared post-COVID-19 patients with healthy young/middle-aged adults [10,11,12,27,29,35,89,91,92,94,96,97,100,102,105,107,108]; however, 13 with middle-aged or older participants matched for age and CV risk factors or ASCVD [10,11,26,28,29,33,93,98,99,101,103,104,106]. One study included a control group with myalgic encephalomyelitis/chronic fatigue syndrome (ME/CFS) [108], and three included patients with non-COVID-19 pneumonia/cardio-respiratory symptoms as the control group [33,90,93]. Additionally, nine studies compared symptomatic post-COVID participants with long-term COVID-19 syndrome (LC) [11,27,28,99,104,105,107,108] with non-LC or non-COVID-19 controls. LC was defined as symptoms persisting three months after diagnosis that last for at least two months without any other explanation [109].

Six studies assessed variability in FMD and nine in cfPWV in post-COVID participants during F/U. Of those, two studies concerned the changes in the endothelial function or AS between the pre-COVID-19 period and the early recovery [101,102], four between the acute/subacute COVID-19 and the early recovery [14,28,91,110], two between the acute/subacute COVID-19 and the mid-term recovery [91,110], six between the early recovery and the mid-term recovery [91,98,102,110], two between the early recovery and the late recovery [28,99], and two between the mid-term recovery and the very late recovery periods [12,35]. Three studies followed post-COVID-19 cohorts with or without an intervention, such as cardiopulmonary rehabilitation or supplement administration [98,111,112]. In the case of these studies, we included only the control groups in the analysis, which did not undergo the intervention.

**Figure 1:**
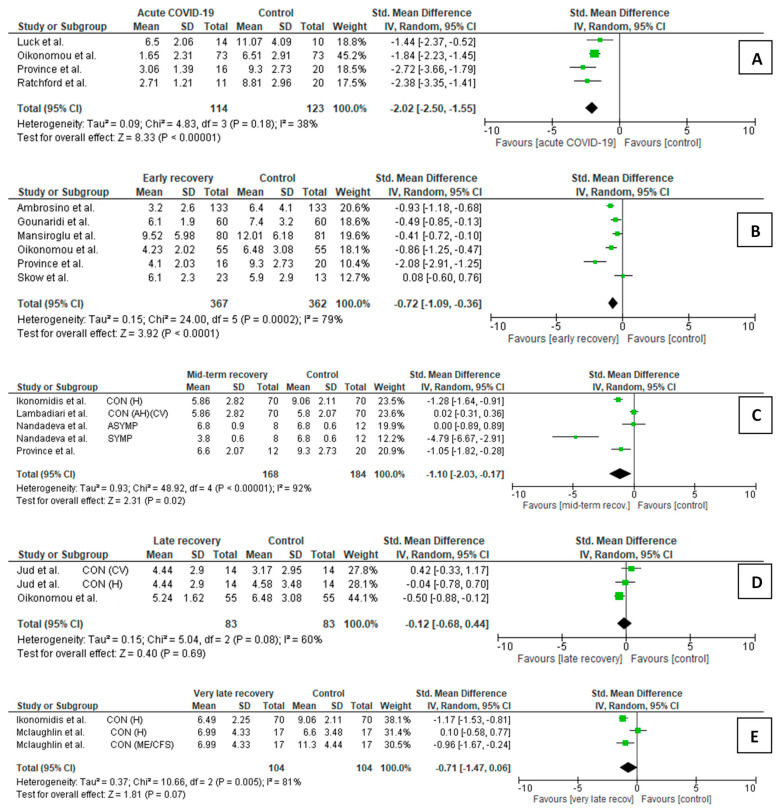
Forest plots for relations in brachial flow-mediated dilation between COVID-19 participants at a different stage of the disease/recovery and controls: (**A**) acute/subacute COVID-19 patients vs. controls [28,89,91,94]; (**B**) early recovery post-COVID-19 patients vs. controls [26,28,91,96,97,98]; (**C**) mid-term recovery post-COVID-19 vs. controls [11,12,27,91]; (**D**) late recovery post-COVID-19 vs. controls [10,28]; (**E**) very late recovery post-COVID-19 vs. controls [12,108]; SD: standard deviation, CI: confidence interval; I^2^: heterogeneity; df: degrees of freedom. Each green square in Figure 1 represents a study’s effect size, and the square’s area represents the magnitude of a related study’s effect size. The lines on either side of the squares indicate the lower and upper limits in a 95% confidence interval (CI) of the calculated effect sizes. The black rhombus at the bottom of the plot shows the calculated overall effect size.

**Figure 2:**
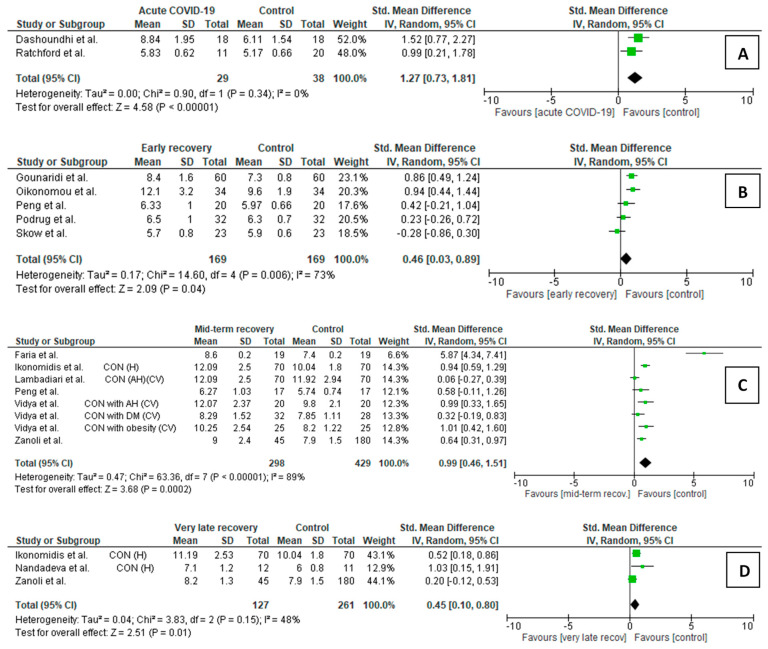
Forest plots for relations in carotid-femoral pulse wave velocity between COVID-19 participants at a different stage of the disease/recovery and controls: (**A**) acute/subacute COVID-19 patients vs. controls [89,92]; (**B**) early recovery patients vs. controls [96,98,99,101,102]; (**C**) mid-term recovery vs. controls [11,12,35,105,106]; (**D**) very late recovery vs. controls [12,35,107]; SD: standard deviation, CI: confidence interval; I^2^: heterogeneity; df: degrees of freedom. Each green square in Figure 2 represents a study’s effect size, and the square’s area represents the magnitude of a related study’s effect size. The lines on either side of the squares indicate the lower and upper limits in a 95% confidence interval (CI) of the calculated effect sizes. The black rhombus at the bottom of the plot shows the calculated overall effect size.

Our meta-analysis was supported by data from 14 FMD studies (627 cases and 694 controls) (Figure 1, Supplementary Figure S2) and 15 cfPWV studies (544 cases and 669 controls) (Figure 2, Supplementary Figure S3).

Table 1 and Table 2 present the study cohorts’ main demographic characteristics. Supplementary Tables S2 and S3 show the clinical data of the included cohorts. Supplementary Tables S4 and S5 describe the study’s design, methodology, main findings, and NOS score.

Our meta-analysis was supported by data from 14 FMD studies (627 cases and 694 controls) (Figure 1, Supplementary Figure S2) and 15 cfPWV studies (544 cases and 669 controls) (Figure 2, Supplementary Figure S3).

### 3.2. Meta-Analysis of Flow-Mediated Dilation

#### 3.2.1. FMD Comparison Between COVID-19 Patients and Controls

The meta-analyses’ results of COVID-19 patients vs. controls were summarized using forest plots (Figure 1A–E).

Acute/subacute COVID-19 participants vs. controls (Figure 1A)

Four studies met the inclusion criteria in this comparison, comprising 114 acute/subacute COVID-19 patients and 123 controls. Acute/subacute COVID-19 patients showed statistically lower mean FMD than the controls (SMD = −2.02, *p* < 0.001).

Early recovery post-COVID-19 participants vs. controls (Figure 1B)

Six studies comparing 367 post-COVID-19 participants in early recovery vs. 362 controls met the inclusion criteria. Pooled analysis showed that, in early recovery, post-COVID-19 patients persisted with a lower mean FMD than the controls (SMD −0.72, *p* < 0.001). However, among the included studies, in one study by Skow et al. [96], patients had slightly higher FMD than the controls.

Mid-term recovery post-COVID-19 participants vs. controls (Figure 1C)

In mid-term recovery, FMD was also lower in post-COVID-19 patients than in controls (SMD = −1.10, *p* = 0.02). For this comparison, three studies (five cohorts) were included. However, a study by Lambadiari et al. [11] had two control groups (healthy individuals and matched controls with arterial hypertension), and in Nandadeva et al.’s study [27], two different groups of COVID-19 patients could be distinguished, i.e., symptomatic and asymptomatic. Sensitivity analysis showed that the SMD lacked significance after excluding the study conducted by Lambadiari et al. [11], containing healthy controls, or Nandadeva et al. [27], with symptomatic COVID-19 convalescents. Thus, the results of this comparison should be treated with caution.

Late recovery post-COVID-19 participants vs. controls (Figure 1D)

Two studies (three cohorts) demonstrated data for post-COVID-19 participants in late recovery compared to controls, including 83 post-COVID-19 patients and the same number of controls. The SMD obtained in this analysis was insignificant (SMD = −0.12, *p* = 0.69), but the sensitivity analysis results were stable.

Very late recovery post-COVID-19 participants vs. controls (Figure 1E)

Still, in two studies (three cohorts) performed in the very late recovery period, post-COVID-19 patients showed similar mean values of FMD to the controls (SMD −0.71, *p* = 0.07). However, this comparison may not be stable because after excluding the subgroup containing healthy controls from the Mclaughlin et al. study [108], the SMD was significant, *p* < 0.001.

The above-mentioned comparisons show no publication bias assessed with Egger’s and Begg’s tests. Table 3 demonstrates the exact results of the publication bias test.

#### 3.2.2. FMD Changes in COVID-19 Patients During Follow-Up

Due to the few studies suitable for meta-analysis, only those assessing changes in FMD between the acute/subacute, the early recovery, and the mid-term recovery periods were compared (Supplementary Figure S2).

In the early recovery vs. acute/subacute COVID-19 period compared to the two studies, a significantly higher FMD was demonstrated in those in early recovery than those with acute/subacute COVID-19 (SMD = 0.95, *p* < 0.001). In turn, three studies showed eligibility in the mid-term recovery vs. early recovery post-COVID-19 period analysis. Between those periods, the FMD still improved (SMD = 0.92, *p* = 0.006). However, the results should be treated with caution due to unstable sensitivity analysis; after excluding Belcaro et al. [111], the difference is insignificant (*p* = 0.09), as well as after excluding Province et al. [91] (*p* = 0.09).

### 3.3. Meta-Analysis of Carotid-Femoral Pulse Wave Velocity

#### 3.3.1. cfPWV Comparison Between COVID-19 Patients and Controls

The meta-analyses’ results of COVID-19 patients and controls were summarized using forest plots (Figure 2A–D).

Acute/subacute COVID-19 vs. controls (Figure 2A)

Only two studies met the inclusion criteria in this comparison: 29 acute/subacute COVID-19 patients and 38 controls. Acute/subacute COVID-19 patients showed statistically higher mean cfPWV than controls (SMD = 1.27, *p* < 0.001).

Early recovery post-COVID-19 participants vs. controls (Figure 2B)

Five studies were included in this comparison, with 169 post-COVID-19 patients and 169 controls. The cfPWV was significantly higher in post-COVID-19 convalescents than in controls (SMD = 0.46, *p* = 0.040). However, one of the studies [96] demonstrated lower cfPWV in convalescents than the controls. In the sensitivity analysis, after subsequent excluding studies by Peng et al. [102], then by Oikonomou et al. [99] and Gounaridi et al. [98], there were no significant differences between the groups (*p* = 0.09, *p* = 0.19, and *p* = 0.19, respectively).

Mid-term recovery post-COVID-19 participants vs. controls (Figure 2C)

Five studies (eight cohorts) met the inclusion criteria. The post-COVID-19 group comprised 298 patients, while the control group comprised 429. In the mid-term recovery period, cfPWV was still higher in post-COVID-19 participants than in the controls (SMD = 0.99, *p* < 0.001). One of the authors, Vidya et al. [106], analyzed three sets of post-COVID-19 and reference participants, i.e., with hypertension, obesity, or diabetes (DM). We observed the lowest SMD when participants with DM were compared (SMD = 0.32). Sensitivity analysis showed that the results are very stable.

No analysis was performed between late recovery patients and controls due to only one study with a late recovery period [99].

Very late recovery post-COVID-19 participants vs. controls (Figure 2D)

In this comparison, three studies demonstrated data on very late recovery after COVID-19 (127 patients and 261 controls). The pooled analysis showed significant SMD between the very late recovery post-COVID-19 patients and controls (SMD = 0.45, *p* = 0.010).

In comparing studies performed in the early recovery period, no publication bias was assessed using Egger’s and Begg’s tests. Compared to studies on the recovery period in the medium term, Egger’s bias test showed significant results; however, they were very close to the bound of significance (*p* = 0.049). After excluding the study by Faria et al. [105] from mid-term recovery vs. controls comparison, no bias between the studies was demonstrated. For the very late recovery vs. controls comparison, after omitting a study by Ikonomidis et al. [12], there was a lack of significance in SMD between the studies (*p* = 0.200). The exact results of the publication bias test are demonstrated in Table 4.

#### 3.3.2. cfPWV Changes in COVID-19 Patients During Follow-Up

Each of the analyses of cfPWV variability over time F/U included only two validated studies (Supplementary Figure S3). It was not possible to perform sensitivity analysis using those comparisons. Therefore, the results should be interpreted with caution.

For early recovery post-COVID-19 vs. pre-COVID-19 period, early recovery post-COVID-19 period vs. acute/subacute COVID-19, and mid-term recovery vs. early recovery post-COVID-19 period, no significant differences in cfPWV were observed (SMD = 0.34, *p* = 0.08, SMD = −0.10, *p* = 0.83, SMD = −0.33, *p* = 0.32, respectively). In the very late recovery vs. mid-term post-COVID-19 period comparison, patients in the very late recovery period had significantly lower cfPWV than those in the mid-term recovery period (SMD = −0.39, *p* < 0.001).

## 4. Discussion

Our meta-analysis of 18 studies showed the most pronounced decrease in FMD compared to controls in the acute/subacute COVID-19, with partial improvement from the acute/subacute phase to early and mid-term recovery and normalization of impaired vascular endothelial function compared to controls in the late recovery period, i.e., >180–270 days after COVID-19 onset. In contrast, cfPWV values, which were higher than controls in the acute/subacute phase, remained elevated throughout the F/U, with no significant changes except for a decrease from mid-term to very late recovery. In the very late recovery, i.e., >270 days after COVID-19 onset, the cfPWV remained higher than that for the controls.

The results of several studies (included in our meta-analysis and others) regarding acute/subacute COVID-19 and early recovery are consistent and usually indicate decreased FMD and increased cfPWV in COVID-19 patients. However, the time and degree of FMD and cfPWV improvement can be influenced by various factors, including the severity of acute COVID-19, the persistence of LC symptoms, as well as the patient’s initial vascular age depending on metric age and CV risk factors or ASCVD, which is discussed below.

In summary, it was observed that FMD deteriorated during acute COVID-19 [66] but started to improve during early recovery in both healthy individuals [91,111] and patients with CV risk factors/ASCVD [28]. Some researchers [27,102] reported that FMD returned to normal in healthy, asymptomatic young adults after experiencing mild COVID-19 during mid-term recovery. However, in patients with a more severe course of COVID-19 [12,28], CV diseases [28], or persistent LC symptoms [108], the parameters may not normalize even in the late/very late recovery period (up to 1.36 [0.51] year of F/U in LC cases).

In healthy individuals, the standard FMD value is approximately 5.7–6.2% for females and 6.7–7.2% for males [113,114]. An FMD value above 7.1% is associated with a lower CV risk, and under 2.9% is associated with a higher risk of major CV events [113]. Such low FMD values (below 2.9%) in young, generally healthy individuals were observed only in the acute/subacute phase of COVID-19 [89], whereas, in middle-aged and elderly individuals with cardiovascular risk factors who were hospitalized due to severe COVID-19 (with COVID-19 pneumonia or treatment in the intensive care unit), they were also in the early stage of convalescence [28,93]. Impaired vasodilation assessed as FMD in the acute/subacute phase of COVID-19 and during convalescence reflects an ongoing microvascular inflammatory response and impaired autoregulation [50]. Conversely, gradual normalization of this parameter may document the healing process and repair of the vascular endothelium.

As for cfPWV, a gradual improvement was observed in young, healthy individuals from the subacute COVID-19 period to mid-term recovery [110]. However, among middle-aged and elderly adults with CV risk factors, there was a noticeable trend of increasing cfPWV from the subacute phase to early recovery [14], followed by persistently elevated cfPWV until the mid-term recovery period [98,112]. A reduction in cfPWV was noted between mid-term and very late recovery; however, the values did not normalize compared to age-, sex-, and CV risk factor-matched controls [99]. More compromised vascular function in the very late recovery period was also found in those with more severe COVID-19 (12 months after diagnosis) [12], or those suffering from LC (median 15 months, range of F/U up to 30 months) [107], even in the absence of ASCVD.

Given the long-lasting vasculitis [4,5,6] and metabolic alterations [42] in patients who have recovered from COVID-19, this population should be considered at a higher risk for ASCVD. Therefore, assessing AS, a marker of vascular aging that seems to accompany those after COVID-19 longer than endothelial dysfunction, can be a valuable option for long-term monitoring in routine medical practice. Risk assessment based on AS may help formulate recommendations for lifestyle changes and decide on earlier pharmacological treatment for ASCVD risk factors such as low-density lipoprotein cholesterol, blood pressure, and prediabetes in otherwise healthy post-COVID-19 individuals. The 2021 European Society of Cardiology (ESC) Guidelines on the prevention of cardiovascular disease in clinical practice [115] and the 2024 ESC Guidelines on the treatment of elevated blood pressure and hypertension [9] both recommend this approach for CV risk stratification and therapeutic decisions. According to these guidelines, a cfPWV higher than 10 m/s indicates an increased CV risk. Values exceeding 10 m/s have been reported from the acute phase of COVID-19 to late/very late convalescence in middle-aged and elderly, apparently healthy individuals hospitalized in the acute phase of the disease [10,11,12] or with symptoms of chronic COVID-19 syndrome [11,100], and in individuals with cardiovascular risk factors [33,99,106]. Tracking changes in this parameter can reflect a reduction in the amount of inflammation, improving arterial elasticity and regulating the renin–angiotensin–aldosterone system (with normalization of blood volume and blood pressure) associated with convalescence after a serious illness. It can also reflect pharmacological and non-pharmacological interventions implemented for ASCVD prevention.

### 4.1. Differences in FMD and cfPWV Following COVID-19 Compared to Healthy Individuals

Some studies compared endothelial function and AS parameters between those who recovered from COVID-19 and healthy age-matched individuals or young adults.

#### 4.1.1. Acute/Subacute COVID-19

Our meta-analysis reviewed four studies comparing FMD in participants with acute/subacute COVID-19 to control groups. This cohort showed lower mean FMD (SMD −2.02, *p* < 0.001). Among these studies, two specifically compared COVID-19 patients in the subacute phase (within 30 days of disease onset) to healthy controls. Province et al. [91] found a mean FMD value of 3.06 (1.39)%, and Ratchford et al. [89] found 2.71 (1.21)%. Both values were markedly lower among young, post-COVID-19 participants compared to the young, healthy controls (9.30 [2.73]% and 8.81 [2.96]%).

Two studies with healthy controls compared cfPWV in acute/subacute COVID-19 patients, revealing a higher mean cfPWV than the controls (SMD = 1.27, *p* < 0.001). In the study by Dashoundhi et al. [92], cfPWV was 8.84 (1.95) m/s in acute COVID-19 and 6.11 (1.54) m/s in healthy controls. In the Ratchford et al. [89] study, cfPWV was still 0.75 m/s higher in the subacute post-COVID-19 individuals than in healthy adults.

#### 4.1.2. Early Recovery Period

Most studies comparing post-COVID-19 participants with controls have focused on early recovery.

Six of those assessing FMD and five assessing cfPWV fit our meta-analysis. In the pooled analysis, FMD in post-COVID-19 participants was persistently lower than the controls (SMD −0.72, *p* < 0.001), although the difference was not as high as in the acute/subacute COVID-19 phase. Two studies (Mansiroglu et al. [97] and Province et al. [91]) compared young post-COVID-19 participants (with or without some CV risk factors) with healthy adults, and the results were consistent with those obtained for the entire group. In the study by Province et al. [91], FMD was lower among post-COVID-19 participants within both the second (4.10 [2.03]%) and third (3.91 [2.64]%) month following a positive SARS-CoV-2 test. However, in the third research work by Skow et al. [96], FMD was not different between participants in early recovery after the Omicron variant of COVID-19 and vaccinated healthy controls. The authors suggested that the Omicron variant does not impact vascular health as significantly as previous variants. Other studies regarding this period, although unsuitable for our meta-analysis, found slight differences between the post-COVID-19 groups and healthy adults. In a pre-post study by Peng et al. [102], FMD in young adults was 10.80 (9.68–11.55)% in post-COVID-19 early recovery and 12.65 (10.30–15.38)% in the pre-COVID-19 period. In turn, in the Luck et al. [94] study, there was only a trend for the difference in FMD when allometrically scaled to account for differences in the baseline brachial artery diameter.

In early recovery, cfPWV was significantly higher in post-COVID-19 convalescents than in the controls (SMD = 0.46, *p* = 0.040) in the whole study set. Contrary, in young, healthy adults in the study by Skow et al. [96], cfPWV did not differ between asymptomatic post-COVID-19 (Omicron variant) participants and vaccinated healthy controls. Similarly, in the Peng et al. [102] young cohort, no difference between measurements performed after mild COVID-19 (6.33 [1.00] m/s) and in the pre-COVID-19 period (5.97 [0.66] m/s) was shown. However, in an additional study by Tudoran et al. [100], cfPWV was higher in post-COVID-19 premenopausal women without metabolic syndrome but with LC (10 [9–11] m/s) than in healthy controls (7 [6,7] m/s).

#### 4.1.3. Mid-Term Recovery Period

When three relevant studies were assessed, it was found that FMD was also lower in post-COVID-19 patients than in controls during the mid-term recovery period (SMD −1.10, *p* = 0.02). Of those, in the research conducted by Lambadiari et al. [11], FMD was lower in the post-COVID-19 healthy participants (37.87% with LC) than in healthy controls (5.86 [2.82]% vs. 9.06 [2.11]%). However, Nandadeva et al. [27] found similar FMD results between the whole post-COVID-19 group compared to healthy participants, albeit not in the LC subgroup. In other research, Faria et al. [105] described 45% lower FMD in post-COVID-19 participants (all with LC) than in healthy controls. Province et al. [91] also observed persistently lower FMD among asymptomatic post-COVID-19 groups compared to the healthy controls in the fourth (4.40 [1.90]%) and even sixth month of recovery (6.60 [2.07]%). However, Peng et al. [102] found no significant difference in FMD between the post-COVID-19 and the pre-COVID-19 period.

On the other hand, cfPWV was higher in patients who have been hospitalized due to COVID-19 than in healthy controls in all of the following included studies: Lambadiari et al. [11] (post-COVID-19: 12.09 [2.50] m/s), Zanoli et al. [35] (post-COVID-19: 9.0 [2.4] m/s), and Faria et al. [105] (post-COVID-19: 8.6 [0.2]), with SMD = 0.99 (*p* < 0.001). However, in studies that were unsuitable for the meta-analysis, namely, those by Nandadeva et al. [27] (which did not provide hospitalization data) and Peng et al. [102] (which focused on non-hospitalized patients), cfPWV was similar in the whole post-COVID-19 group compared to controls.

#### 4.1.4. Late Recovery Period

The SMD obtained for the difference in FMD between post-COVID-19 and controls during the late recovery was insignificant (SMD = 0.12, *p* = 0.69). In the one analysis by Jud et al. [10], FMD values were similar between elderly post-COVID-19 participants (4.44 [2.90]%) and healthy controls (4.58 [3.48]%).

Due to the limited data (only one study by Oikonomou et al. [99] concerning a late recovery CV participants), no cfPWV analysis was performed comparing late recovery patients with controls. In the non-included study by Jud et al. [10], cfPWV was higher in the elderly post-COVID-19 participants (10.75 [8.10–11.45] m/s than in only sex-matched younger, healthy controls (5.70 [5.38–6.05] m/s). However, the results did not differ when comparing the post-COVID-19 participants and age- and sex-matched controls with ASCVD.

#### 4.1.5. Very Late Recovery Period

In the Ikonomidis et al. [12] study (healthy, middle-aged participants, with only 4.25% having LC) and the Mclaughlin et al. [108] study (middle-aged participants, all with LC), FMD values remained impaired in post-COVID-19 very late recovery groups (6.49 [2.25]% and 6.99 [4.33]%) compared to healthy control groups. However, when the ME/CSF control group was added from the Mclaughlin et al. [108] study, the result of the meta-analysis was insignificant (SMD −0.71, *p* = 0.07). Additional studies did not bring a straightforward solution: in Gao et al.’s [29] research, FMD remained lower in middle-aged post-COVID-19 patients with ASCVD and LC (3.5 [2.2–4.6]%) than in healthy controls; however, in the study by Nandadeva et al. [107], FMD was not different between the young LC group not burdened with CV diseases and young healthy controls.

What is concerning regarding cfPWV is that the pooled analysis results showed significant SMD between very late recovery post-COVID-19 patients and controls (SMD = 0.45, *p* = 0.010). Both the Ikonomidis et al. [12] (post-COVID-19: 11.19 (2.53) m/s) and the Nandadeva et al. [107], (LC group: 7.1 [1.2] m/s) studies were consistent with this result. In the study by Zanoli et al. [35], comparing cfPWV between middle-aged post-COVID-19 individuals and age-, sex-, and BMI-matched healthy controls, the difference was no longer visible. The authors noticed that the improvement in cfPWV was negatively associated with the interval from the acute disease onset to measurement.

### 4.2. Differences in FMD and cfPWV Following COVID-19 Compared to Particpiants with Cardiovascular Risk Factors or Atherosclerotic Cardiovascular Diseases

In general, individuals with CV risk factors or ASCVD have impaired endothelial function and increased AS [51,62]. Persistent low-grade inflammation, dysregulation of the renin–angiotensin system, insulin resistance, and increased tissue stiffness in these patients can lead to an overexpression of pro-inflammatory cytokines in response to COVID-19. It can disrupt endothelial integrity, facilitating viral entry into tissues and enhancing platelet adhesion and activation, which may result in thrombus formation and the destabilization of atherosclerotic plaques [116,117,118,119]. The above factors may contribute to a more severe course of acute COVID-19 [118,119,120] and increased or prolonged vascular dysfunction in the post-COVID-19 period. Consequently, the study and control groups with CV risk factors/ASCVD might exhibit lower FMD and higher cfPWV than healthy adults, affecting the results of comparative analyses [11,26,100,106]. In the following sections, we discuss studies that compared COVID-19 recoveries with CV risk factor-matched control groups.

#### 4.2.1. Acute/Subacute COVID-19

Of the studies included in the meta-analysis, only Oikonomou et al. [28] assessed the difference in FMD between middle-aged or elderly participants with CV risk factors in the acute COVID-19 phase and the propensity score-matched controls. They showed that FMD is impaired in the acute COVID-19 phase (1.65 [2.31]%) compared to CV controls (6.51 [2.91]%). In a non-included study by Ciacci et al. [93], FMD was also lower in COVID-19 elderly patients (2.1 [0–5.7]) than in the age- and CV risk factor-matched controls (5.6 [4.7–7.4]). This indicates an additive effect of COVID-19 on ASCVD-induced vasculopathy.

#### 4.2.2. Early Recovery Period

During the early recovery period, three analyzed studies accessed FMD in middle-aged or elderly post-COVID-19 participants compared to age-, sex- and CV risk factor-matched controls. In the study by Ambrosino et al. [26], FMD was impaired in post-COVID-19 participants (3.2 [2.6]%). Furthermore, recent COVID-19 infection emerged as an independent predictor of FMD values (β = −0.427, *p* < 0.001). However, when more than three concomitant CV risk factors were present, the difference in FMD values between cases and controls was no longer significant, likely due to considerable deterioration in FMD among the controls, attributed to vascular aging and CV comorbidities. In the study by Oikonomou et al. [28], FMD remained reduced and amounted to 4.23 (2.02)%. Also, in the study by Gounaridi et al. [98], the result was consistent. In another study by Ergül et al. [95], COVID-19 (odds ratio [OR] 3.611, 95% CI 1.069–12.198, *p* = 0.039) and BMI (OR 1.122, 95% CI 1.023–1.231, *p* = 0.015) were independent predictors of endothelial dysfunction.

Similarly, three studies within a meta-analysis comparing cfPWV to CV controls unanimously indicated a persistent increase in AS in the early post-COVID-19 period. In a study by Gounaridi et al. [98], cfPWV was 8.4 (1.6) m/s in the post-COVID-19 group (vs. 7.3 [0.8] m/s); in Oikonomou et al.’s study [99], cfPWV was 12.1 (3.2) m/s (vs. 9.6 [1.9] m/s, *p* < 0.001); and in Podrug et al.’s study [101], cfPWV was 6.5 (1.0) m/s (vs. 6.3 [0.7] m/s). In an additional study by Tudoran et al. [100], altered cfPWV correlated, among others, with the time elapsed since COVID-19 diagnosis (r = −0.66, *p* < 0.0001) and a number of factors associated with metabolic syndrome (r = 0.41, *p* < 0.0001).

#### 4.2.3. Mid-Term Recovery Period

Among the analyzed studies performed in mid-term recovery, only Lambadiari et al. [11] assessed FMD compared to the CV control group. FMD was similar between middle-aged post-COVID-19 participants with a single ASCVD risk factor, i.e., hypertension (5.86 [2.82]%) compared to hypertensive controls (5.80 [2.07]%). However, in an additional study by Riou et al. [103], FMD was lower in middle-aged post-COVID-19 patients with several ASCVD risk factors compared to age- and sex-matched controls. In the study, up to 44% of post-COVID-19 convalescents presented with a reduced FMD (< 8%) three months after hospitalization.

Concerning cfPWV, in the Vidya et al. [106] study, cfPWV was higher among the post-COVID-19 group with hypertension (12.07 [2.37] m/s) and the post-COVID-19 with obesity (10.25 [2.54] m/s) when compared to risk-factor matched controls (9.80 [2.10] m/s and 8.20 [1.22] m/s, respectively). However, cfPWV did not differ in patients with DM between those after COVID-19 (8.29 [1.52] m/s) and the controls (7.85 [1.11] m/s). cfPWV was the highest in the post-COVID-19 group with hypertension, followed by the post-COVID-19 with obesity and the post-COVID-19 with DM populations.

#### 4.2.4. Late Recovery Period

In the late recovery period (approximately seven months after COVID-19), the two analyzed studies gave divergent results regarding the difference in FMD between the middle-aged or elderly post-COVID-19 participants with CV risk factors/ASCVD and ASCVD controls. Oikonomou et al. [28] still observed impaired FMD (post-COVID-19: 5.24 [1.62]% vs. controls: 6.48% [3.08], *p* = 0.01); however, Jud et al. [10] found no such difference (post-COVID-19: 4.44 [2.90]% vs. controls: 3.17 [2.95]%, *p* ≥ 0.05).

Researchers received similar results for cfPWV. In the Oikonomou et al. [99] study, post-COVID-19 individuals presented with impaired cfPWV compared to controls (11.7 [2.7] m/s vs. 9.6 [1.9] m/s, *p* < 0.001); however, in Jud et al.’s [10] study, cfPWV was similar between groups (post-COVID-19: 10.75 [8.10–11.45] m/s vs. controls: 9.95 [8.40–11.60] m/s, *p* ≥ 0.05).

#### 4.2.5. Very Late Recovery Period

In the very late recovery period, only the study by Gao et al. [29] was performed, and impaired FMD in post-COVID-19 convalescents compared to risk-matched controls was found (post-COVID-19: 3.5 [2.2–4.6]% vs. controls: 7.7 [5.1–10.7]%).

None of the studies compared cfPWV in the post-COVID-19 groups vs. CV controls in the very late recovery period.

### 4.3. Associations of Differences in FMD and cfPWV with Age

Vascular wall abnormalities increase with age, and this is partially dependent on the number of CV risk factors and comorbidities [51,58]. Both arteriosclerosis and atherosclerosis contribute to vascular changes by increasing the stiffness of blood vessels and reducing their ability to buffer pulsatile arterial blood flow. A key characteristic of arteriosclerosis is the loss of elastin fibers, which are replaced by collagen in the vessel tunica media, resulting in decreased elasticity. In contrast, atherosclerosis is characterized by the formation of calcified plaques, driven by chronic inflammation that accumulates smooth muscle cells, lipids, connective tissue, and calcium within the intima of large and medium-sized arteries [121]. On the other hand, the age-related reduction in NO bioavailability impairs endothelium-dependent vasodilation with aging [122]. Vascular aging may influence FMD and cfPWV values regardless of COVID-19.

Studies by Province et al. [91], Ratchford et al. [89], Schnaubelt et al. [33], and Dashoundhi et al. [92] confirmed that FMD and cfPWV were impaired even among young (up to the age of 30) individuals with acute/subacute COVID-19 compared to the young, healthy controls. Also, in middle-aged (40–60 years old) or elderly (above 60 years of age) COVID-19 participants, the FMD and cfPWV abnormalities were not only age-dependent, which is confirmed in studies with age and CV risk factor-matched control groups by Oikonomou et al. [28], Ciacci et al. [93], and Oliveira et al. [90] (for FMD comparisons) and by Schnaubelt et al. [33] (for cfPWV comparisons).

Also, during the convalescence period, many studies documented the worsening of FMD [26,28,98,103] and cfPWV [98,99,101,104,106] in post-COVID-19 participants even when compared to age- and sex-matched controls.

In addition, few studies reported the results of multivariate analyses assessing age as an independent risk factor of cfPWV abnormalities. Kumar et al. [34] found that age (*p* < 0.0001), weight (*p* = 0.0178), and brachial mean arterial pressure (*p* < 0.0001) significantly contribute to cfPWV, apart from the severity of acute COVID-19. In the study by Podrug et al. [101]), age (*p* = 0.005), and time since acute COVID-19 (*p* = 0.030) were positively associated with the cfPWV change in the recovery period. In the study by Zanoli et al. [35], age (*p* = 0.005), time from COVID-19 onset (*p* = 0.045), high-sensitivity C-reactive protein at hospitalization due to COVID-19 (*p* = 0.04), and mean blood pressure (*p* = 0.01) were independently associated with cfPWV.

It is worth considering whether pre-existing metrics and, more importantly, vascular age may influence the persistence of post-COVID-19 vasculopathy, as described in Section 4.4.

### 4.4. Associations of Changes in FMD and cfPWV with Time Since COVID-19 Onset

#### 4.4.1. Changes Observed in Young and Middle-Aged Healthy Adults

Walia et al. [66] compared FMD values before and after seven days of the disease onset and found that endothelial function deteriorated during the acute phase of COVID-19 in healthy middle-aged adults. In acute COVID-19 (Province et al. [91] and Ratchford et al. [89]) and early recovery (Mansiroglu et al. [97], Province et al. [91], Peng et al. [102]), young patients recovered from COVID-19 were characterized by impaired endothelial function, which was assessed using FMD, compared to healthy adults. Later on, FMD started to improve from 7.2 (1.0)% to 8.0 (0.9)% during the early recovery and then to 8.8 (1.4)% during the mid-term recovery period, which was described by Belcaro et al. [111]. Also, Peng et al. [102], observing healthy young adults after mild COVID-19, found an improvement in FMD from the early recovery (10.80 [9.68–11.55]%) to the mid-term recovery period (11.10 [10.30–11.35]%), with its normalization compared to the pre-COVID-19 period over 3.8 months since COVID-19 diagnosis. Nandadeva et al. [27] and Nandadeva et al. [107] also found full normalization of FMD in young post-COVID-19 participants compared to controls in the mid-term recovery period in asymptomatic individuals and in the very late recovery period in symptomatic (with LC) individuals.

However, Province et al. [91] did not find an increase in FMD from the subacute COVID-19 phase until the sixth month of mid-term recovery (Hedge’s g = −1.737). Moreover, in the sixth month of recovery, FMD persistence decreased compared to healthy controls. FMD was still also abnormal in the study by Faria et al. [105]. Moreover, Lambadiari et al. [11] and Ikonomidis et al. [12], in a combined study, confirmed that FMD may not improve until the very late recovery period and remains lower than in healthy controls at the 12-month point after infection. Similar results were obtained by Mclaughlin et al. [108] in an LC cohort at 1.36 (0.51) years from disease onset.

The pre-post study by Peng et al. [102] compared cfPWV between pre-COVID-19 and post-COVID-19 periods and did not find any increase in cfPWV in young, healthy adults after mild COVID-19, neither in early nor in mid-term recovery. However, in the study by Ratchford et al. [89], cfPWV was higher in the subacute COVID-19 group than in young, healthy controls. Then, Szeghy et al. [110] showed a decrease in cfPWV from the subacute COVID-19 period (5.70 [0.73] m/s) to the mid-term recovery period (at the sixth month of F/U: 4.88 [0.65] m/s). Despite this, in mid-term recovery, cfPWV was higher in middle-aged, post-COVID-19 patients than in age-, or age- and sex-matched controls in most studies, i.e., Lambadiari et al. [11], Faria et al. [105], and Zanoli et al. [35] (Study 1). Zanoli et al. [35] (Study 2) observed that cfPWV improved by 9% from mid-term recovery (8.8 [2.0] m/s) to the very late recovery period (8.0 [1.6] m/s); however, by the end, the F/U values were still higher than those in the matched controls. On the contrary, in the combined study by Lambadiari et al. [11] and Ikonomidis et al. [12], cfPWV remained increased in the very late recovery as in the mid-term recovery period (11.19 [2.53] m/s vs. 12.09 [2.50] m/s) and impaired compared healthy controls. Similarly, in the Nandadeva et al. [107] cohort of middle-aged individuals with LC syndrome, cfPWV was higher in post-COVID-19 participants compared to age- and BMI-matched controls even in the very late recovery period.

#### 4.4.2. Changes Observed in Middle-Aged and Elderly Participants with CV Risk Factors or ASCVD

In middle-aged participants with acute/subacute COVID-19 and CV risk factors or ASCVD, Oikonomou et al. [28] and Ciacci et al. [93] consistently reported lower FMD than in age- and CV risk factor-matched control groups. Oikonomou et al. [28] reported an increase in FMD from the acute phase (1.75 [2.19]%) to the early recovery (1 month after hospital discharge: 4.23 [2.02]%). However, in the early recovery, three studies—Oikonomou et al. [28], Ambrosino et al. [26], and Gounaridi et al. [98]—confirmed persistently reduced FMD in post-COVID-19 participants compared to controls. Ambrosino et al. [26] found recent COVID-19 as an independent predictor of FMD values (β = −0.427, *p* < 0.001) in early recovery. Then, Gounaridi et al. [98] confirmed an increase in FMD from the early recovery to the mid-term recovery period (5.9 [2.2]% to 6.6 [1.8]%). In mid-recovery, FMD was still abnormal compared to age- and sex-matched controls in the Riou et al. [103] study. Oikonomou et al. [28], continuing observations, described a further increase in FMD from the mid-term to the late recovery period (6 months after hospital discharge: 5.24 [1.62]%) but without its normalization at the end of the F/U. In post-COVID-19 patients with ASCVD and LC, FMD remained lower than age- and risk-matched controls until very late recovery (for example, see Gao et al. [29]).

In the pre-post study, Podrug et al. [101] showed an increase of 0.19 m/s (95% CI -0.04 to 0.41) from the pre-COVID-19 period (6.3 [0.7] m/s) to early recovery (6.5 [1.0] m/s) in middle-aged adults with CV risk factors. Time since acute COVID-19 was positively associated with the cfPWV change. In the study by Schnaubelt et al. [33], cfPWV was higher in the acutely ill elderly patients with cardiorespiratory symptoms and COVID-19 than controls. A slight upward trend in cfPWV, from subacute COVID-19 (11 [3] m/s) to early recovery (12 [3] m/s), was observed by Saloň et al. [14]. In early recovery, two studies evaluated participants aged over 40, i.e., Gounaridi et al. [98] and Oikonomou et al. [99], and confirmed increased cfPWV in the post-COVID-19 groups compared to age- and sex-matched controls. Then, no improvement until the mid-term recovery was noticed by Gounaridi et al. [98] (8.9 [1.8] m/s vs. 8.8 [1.9] m/s in the non-CR group) and Teixeira DO Amaral et al. [112], (*p* = 0.043 in the non-CR group). A decrease was only recorded by Oikonomou et al. [98] from the early recovery (12.1 [3.2] m/s) to the late recovery period (11.7 [2.7] m/s). However, in the post-COVID-19 group, cfPWV remained impaired compared to controls even in late recovery.

### 4.5. Associations of Differences and Changes in FMD and cfPWV with the Severity of the Acute Phase of COVID-19

In the acute phase of COVID-19, hematological, biochemical, inflammatory, and cardiac biomarkers reflect disease severity and predict poor short-term prognosis [123,124,125]. During recovery, up to 12–16 months after acute illness, several to several dozen of those recovered from COVID-19 still had elevated inflammation, hypercoagulability, and vascular damage biomarkers [7,10,23,24,25,29]. This may indicate the persistence of endothelial dysfunction and contribute to increased AS [34,35,100].

Endothelial function impairment was strongly expressed in severely ill patients [28]. FMD reduction ≤ 3.135% (in the Güz et al. [67] study), ≤3.43% (in the Oliveira et al. [90] study), or <4.4% (in the Bianconi et al. [68] study) predicted mortality, intensive care unit (ICU) admission, or prolonged hospital stay. Lower FMD% was also associated with higher lung parenchymal involvement [66,67] and with biomarkers abnormalities, such as elevated levels of D-dimer (r = −0.52, *p* < 0.001), troponin (r = −0.45, *p* < 0.001), ferritin (r = −0.47, *p* < 0.001), lactate dehydrogenase (r = −0.49, *p* < 0.001), and white blood cells count (r = −0.23, *p* = 0.024) [67].

The impact of COVID-19 severity on FMD continued into the recovery period. In the Oikonomou et al. [28] study, FMD was significantly lower in ICU-treated individuals compared to those treated in the medical ward starting from the acute phase (0.48 [1.01]% vs. 2.33 [2.57]%) through early recovery (2.29 [0.86]% vs. 4.63 [1.96]%) until the late recovery period (3.18 [0.69]% vs. 5.67 [1.41]%). Also, in the study by Santoro et al. [69], COVID-19 severity was associated with a 1.354 increased risk of endothelial dysfunction in the mid-term recovery period; lower FMD was more frequent in hospitalized patients (78.3%) than in home-care participants (21.7%). In early (Ambrosino et al. [70]) to mid-term recovery (Santoro et al. [69]), FMD showed a direct correlation with the severity of pulmonary impairment assessed as arterial oxygen tension (*p* = 0.004), forced expiratory volume in 1 s (*p* < 0.01), forced vital capacity (rho = 0.406, *p* < 0.001), and diffusing capacity for carbon monoxide (*p* = 0.008).

Kumar et al. [34] and Schnaubelt et al. [33] documented the correlation between COVID-19 severity and AS assessed as cfPWV. The cfPWV gradually increased with COVID-19 severity, was higher among COVID-19 fatalities than in survivors (*p* = 0.056), and correlated with the duration of hospital stay (*p* = 0.019). The cfPWV measured in the early recovery still correlated, among others, with lung injury (*p* < 0.0001) (Tudoran et al. [100]).

However, the analyzed publications did not report the long-term relationship between hard endpoints, such as mortality, and FMD or cfPWV in the post-COVID-19 period. The ongoing CARTESIAN study [126] aims to provide insights into the risk of CV events related to accelerated vascular aging following SARS-CoV-2 infection. Both measurements, cfPWV and FMD, are planned in participants, along with an assessment of mortality causes and hospitalization data. With a clinical F/U period ranging from 5 to 10 years, this study is designed to yield valuable data on the topic.

### 4.6. Associations of Differences and Changes in FMD and cfPWV with Long-Term COVID-19 Syndrome

LC is a multisystemic disorder that manifests through a variety of symptoms, including fatigue, dyspnea, chest pain, cognitive impairment, sleep disturbances, depression, and others. These symptoms lead to a decline in functional abilities and quality of life, affecting more than 80% of patients [127,128,129,130]. While the LC symptoms tend to decrease over time, over 40% of patients experience them a year after the disease onset [131]. Persistent vasculopathy and low-grade inflammation resulting from COVID-19 may be linked to the symptoms observed in LC syndrome [132,133]. Several authors evaluated the relationship between the presence of LC and FMD or cfPWV abnormalities.

In the mid-term recovery, Nandadeva et al. [27] showed that FMD persisted impaired only in young adults with LC (3.8 [0.6]%), while in the non-LC group (6.8 [0.9]%), it was already normalized compared to healthy controls. Lambadiari et al. [11] found decreased FMD in the whole group of middle-aged recovered patients; however, the values were lower in the LC subgroup than in the non-LC participants (5.99 [2.43]% vs. 4.99 [5.14]%). In Nandadeva et al.’s [107] cohort, regarding very late recovery, there were no longer any differences between the LC and the non-LC groups. On the contrary, Mclaughlin et al. [108] confirmed lower FMD in the middle-aged LC group (6.99 [4.33]%) compared to healthy controls, even in the very late recovery period. What is worth emphasizing is that FDM in post-COVID-19 symptomatic convalescents was similar to FMD in patients complaining of ME/CFS. In Oikonomou et al.’s [28] cohort of middle-aged adults with CV risk factors/ASCVD (58% with LC), FMD was not normalized in late recovery. However, it was probably not related to LC as there were no differences in FMD according to the presence or absence of symptoms (4.98 [1.90]% vs. 5.02 [1.15]%).

In the study by van der Sluijs et al. [104], performed in the mid-term recovery, cfPWV differed only between the LC subgroup of 31 participants and controls, but not in the whole (LC + non-LC) group of 97 participants. In Lambadiari et al.’s [11] study, cfPWV was higher in symptomatic (LC) than in asymptomatic participants (12.27 [2.95] m/s vs. 11.28 [3.11] m/s). In addition, Zanoli et al. [35], in middle-aged healthy adults (63% with LC in mid-term, 58% with persistent symptoms in very late recovery), noticed that the higher the number of persistent symptoms reported during the study, the higher the cfPWV was (*p* = 0.001). In the very late recovery period, the middle-aged, healthy LC cohort evaluated by Nandadeva et al. [107] still had higher cfPWV than the controls. Nevertheless, cfPWV did not correlate with a total symptom burden. On the contrary, Oikonomou et al. [99] found no differences in cfPWV between post-COVID-19 participants with and without LC symptoms in late recovery.

### 4.7. Study Limitations

The number of studies assessing FMD or cfPWV in predefined periods from the onset of COVID-19 that were suitable for statistical analysis was limited. Also, the size of the studied groups was usually small. In addition, devices from different manufacturers were used, which could also result in minor differences in mean values and different measurement errors. A part of the heterogeneity in the results could also be related to the different SARS-CoV-2 variants involved. Most of the study participants from the COVID-19 groups became infected in 2020–2021 when the original Alpha to Gama SARS-CoV-2 variants were dominant [134]. However, some researchers conducting studies in 2022, such as Gounaridi et al. [98] and Skow et al. [96], reported the Omicron variant as dominant. Furthermore, multiple factors, such as age, CV risk, COVID-19 severity, and the presence of LC, were confounded in individual study cohorts influencing outcomes, making it difficult to interpret and compare results across studies.

The limited number of available studies did not allow for reliable subgroup analyses, i.e., studies comparing post-COVID patients with healthy (young/middle-aged) adults, studies comparing post-COVID patients with controls matched for age and CV risk factors or ASCVD, or studies comparing post-COVID patients with LC symptoms with non-LC or healthy controls. Considering the small number of studies, we lowered the allowable NOS score from seven points planned during protocol registration in PROSPERO to six points to avoid rejecting some studies with a controlled group. In addition, some sensitivity analyses are unstable or not feasible because only two studies were included. Therefore, the results should be considered preliminary and require repetition if more publications are available.

## 5. Conclusions

Increasingly, long-term observations indicate that vascular function may not improve within 1.5 years after COVID-19. The time to achieve this improvement and whether the improvement is complete or partial may depend on the severity of acute COVID-19, the persistence of LC symptoms, age, and pre-existing CV risk factors. Persistent COVID-19 vasculopathy could have potential implications for vascular aging and ASCVD risk.

## Figures and Tables

**Table 1:** Demographic data of cohorts included in studies comparing post-COVID-19 participants and non-COVID-19 controls.

Study	Country	Compared Study Groups	Age (SD)	Sex M/F
Ambrosino et al. [26], 2021	Italy	133 post-COV in early recovery (non-CR)	61.6 (10.6)	108M/25F
		133 CON (CV)	60.4 (11.5)	107M/26F
Ciacci et al. [93], 2023	Italy	20 acute COV	70 (17)	14M/6F
		20 CON (CV)	74 (5)	15M/5F
		20 CON (PN)	71 (16)	12M/8F
Dashoundhi et al. [92], 2023	India	18 acute COV	31.83 (9.75)	7M/11F
		18 CON (H)	30.61 (10.11)	7M/11F
Ergül et al. [95], 2022	Turkey	63 post-COV in early recovery	44.4 (14.4)	-
		29 CON		
Faria et al. [105], 2023	Brazil	19 post-COV in mid-term recovery (LC)	47.0 (8.0)	12M/7F
		19 CON (H)	43.0 (10.0)	11M/8F
Gao et al. [29], 2022	China	86 post-COV in very late recovery (LC)	58 (39–70)	32M/54F
		28 CON (H)	56 (37–65)	10M/18F
		30 CON (CV)	62 (39–67)	11M/19F
Gounaridi et al., 2023 [98]	Greece	60 post-COV in early recovery	52.2 (12.3)	29M/31F
		60 CON (CV)	55.3 (9.8)	32M/28F
Ikonomidis et al. [12], 2022	Greece	70 post-COV in very late recovery	54.53 (9.07)	44M/26F
		70 CON (H)	54.77 (8.95)	44M/26F
Jud et al. [10], 2021	Austria	14 post-COV in late recovery	68.7 (12.0)	7M/7F
		14 CON (H)	30.7 (4.2)	7M/7F
		14 CON (CV)	66.9 (10.9)	7M/7F
Lambadiari et al. [11], 2021	Greece	70 post-COV in mid-term recovery (LC)	54.53 (9.07)	44M/26F
		70 CON (H)	54.77 (8.95)	44M/26F
		70 CON (CV)	54.47 (8.83)	44M/26F
Luck et al. [94], 2023	Pennsylvania, US	14 subacute COV	20 (1)	10M/4F
		10 CON (H)	22 (2)	7M/3F
Mansiroglu et al. [97], 2022	Turkey	80 post-COV in early recovery	32.10 (5.87)	32M/48F
		81 CON (H)	30.51 (7.33)	36M/45F
Mclaughlin et al. [108], 2023	Scotland, UK	17 post-COV in very late recovery (LC)	47.52 (9.60)	4M/13F
		17 CON (H)	49.05 (13.77)	7M/10F
		17 CON (ME/CFS)	49.7 (9.78)	7M/10F
Nandadeva et al. [27] 2021	Texas, US	8 post-COV in mid-term recovery (non-LC)	22 (4)	5M/3F
		8 post-COV in mid-term recovery (LC)	24 (3)	1M/7F
		12 CON (H)	23 (3)	6M/6F
Nandadeva et al. [107] 2023	Texas, US	12 post-COV in very late recovery (LC)	48 (9)	0M/12F
		11 CON (H)	50 (13)	0M/11F
Oikonomou et al. [28], 2022	Greece	73 acute COV	60.0 (12.7)	46M/27F
		73 CON (CV)	62.9 (14.0)	49M/24F
		55 post-COV in early recovery	57.8 (12.7)	32M/23F
		55 post-COV in late recovery (LC)	57.8 (12.7)	32M/23F
		55 CON (CV)	62.6 (16.1)	29M/21F
Oikonomou et al. [99], 2023	Greece	34 post-COV in early recovery	57.2 (12.9)	26M/8F
		30 post-COV in late recovery (LC)	-	-
		34 CON (CV)	57.4 (12.8)	23M/11F
Oliveira et al. [90], 2021	Brazil	98 acute COV	61 (16)	55M/43F
		82 CON (PN)	63 (17)	40M/42F
Province et al. [91], 2022	North Carolina, US	16 subacute COV	21 (1.0)	8M/8F
		16 post-COV in early recovery		
		12 post-COV in mid-term recovery	21 (1.0)	7M/5F
		20 CON (H)	23 (1.0)	5M/15F
Ratchford et al. [89], 2021	North Carolina, US	11 subacute COV	20.2 (1.1)	4M/7F
		20 CON (H)	23.0 (1.3)	5M/15F
Riou et al. [103], 2021	France	27 post-COV in mid-term recovery	57 (49–66)	17M/10F
		9 CON (CV)	59 (54–62)	5M/4F
Schnaubelt et al. [33], 2021	Austria	22 acute COV	76.5 (67.0–84.0)	11M/11F
		22 CON (PN) (CV)	76.5 (67.0–83.0)	10M/12F
Skow et al. [96], 2022	Texas, US	23 post-COV in early recovery	23 (3)	9M/14F
		13 CON (H)	26 (4)	6M/7F
Tudoran et al. [100], 2023	Romania	54 post-COV in early recovery (non-MS)	47.76 (5.43)	0M/54F
		67 post-COV in early recovery (MS)	50.59 (4.53)	0M/54F
		40 CON (H)	49.47 (5.14)	0M/54F
van der Sluijs et al. [104], 2023	The Netherlands	31 post-COV in mid-term recovery (LC)	58 (51–63)	17M/14F
		31 CON	57 (50–62)	17M/14F
		97 post-COV in mid-term recovery	-	-
		49 CON	-	-
Vidya et al. [106], 2023	India	IA: 32 post-COV in mid-term recovery with DM	30–50	-
		IB: 28 CON with DM (CV)		
		IIA: 20 post-COV in mid-term recovery with AH		
		IIB: 20 CON with AH (CV)		
		IIIA: 25 post-COV in mid-term recovery with obesity		
		IIIB: 25 CON with obesity (CV)		
Zanoli et al. [35] (Study 1), 2022	Italy	45 post-COV in mid-term recovery (LC)	55 (11)	25M/20F
		45 post-COV in very late recovery	54 (13)	27M/18F
		180 CON (H)	55 (13)	97M/83F

CV: participants with cardiovascular risk factors or atherosclerotic cardiovascular diseases; H: healthy adults; LC: ≥ 30% of participants in cohort with long term-COVID-19 syndrome; ME/CFS: participants with myalgic encephalomyelitis/chronic fatigue syndrome; MS: participants with metabolic syndrome; non-LC: asymptomatic participants; non-MS: participants without metabolic syndrome; PN: participants with non-COVID-19 pneumonia or respiratory symptoms; COV: COVID-19; F: female; M: male.

**Table 2:** Demographic data of cohorts in studies assessing changes in selected parameters during follow-up.

Study	Country	Study Group in F/U	Age (SD)	Sex M/F
Belcaro et al. [111], 2022	Italy	30 post-COV in the early recovery period (non-Pycnogenol^®^ group)	35–70	-
		30 post-COV in the mid-term recovery period (non-Pycnogenol^®^ group)		
Gounaridi et al. [98], 2023	Greece	30 post-COV in the early recovery period (non-CR group)	49.10 (12.70)	18M/12F
		30 post-COV in the mid-term recovery period (non-CR group)		
Lambadiari et al. [11], 2021 Ikonomidis et al. [12], 2022	Greece	70 post-COV in the mid-term recovery period	54.53 (9.07)	44M/26F
		70 post-COV in the very late recovery period		
Oikonomou et al. [28], 2022	Greece	55 in the acute COV phase	57.8 (12.7)	32M/23F
		55 post-COV in the early recovery period		
		55 post-COV in the late recovery period		
Oikonomou et al. [99], 2023	Greece	34 post-COV in the early recovery period	57.2 (12.9)	26M/8F
		34 post-COV in the late recovery period		
Peng et al. [102], 2024	China	37 in the pre-COV period	21.35 (1.99)	27M/10F
		20 post-COV in the early recovery period		
		17 post-COV in the mid-term recovery period		
Podrug et al. [101], 2023	Croatia	32 in the pre-COV period	36.6 (12.6)	18M/14F
		32 post-COV in the early recovery period		
Province et al. [91], 2022	North Carolina, US	16 in the subacute COV phase	21 (1.0)	8M/8F
		16 post-COV in the early recovery period		
		12 post-COV in the mid-term recovery period		
Saloň et al. [14], 2023	Norway	35 in the subacute COV phase	60 (10)	30M/5F
		35 post-COV in the early recovery period		
Szeghy et al. [110], 2022	North Carolina, US	14 in the subacute COV phase	21 (1.0)	7M/7F
		14 post-COV in the early recovery period	21 (1.0)	7M/7F
		12 post-COV in the mid-term recovery period	21 (1.0)	7M/5F
Teixeira DO Amaral et al. [112], 2022	Brazil	20 post-COV in the early recovery period (non-CR group)	53.30 (11.60)	8M/12F
		20 post-COV in the mid-term recovery period (non-CR group)		
Zanoli et al. [35] (Study 2), 2022	Italy	41 post-COV in the mid-term recovery period	54 (12)	21M/20F
		41 post-COV in the very late recovery period		

non-CR group: patients non participated in cardiopulmonary rehabilitation; non-Pycnogenol^®^ group: patients not receiving the supplement; COV: COVID-19; F: female; M: male.

**Table 3:** The results of Egger’s and Begg’s tests for all comparisons between the studies analyzing brachial flow-mediated dilation between COVID-19 participants at a different stage of the disease/recovery and controls.

Comparison	Egger’s Test			Begg’s Test	
	Intercept	95% CI	*p*	Kendall’s Tau	*p*
Acute/subacute COVID-19 patients vs. controls	−1.306	−9.058 to 6.445	0.544	−0.333	0.497
Early recovery post-COVID-19 patients vs. controls	−0.563	−8.732 to 7.605	0.858	−0.067	0.851
Mid-term recovery post-COVID-19 vs. controls	−3.562	−14.758 to 7.634	0.386	−0.400	0.327
Late recovery post-COVID-19 vs. controls	3.912	−16.060 to 23.884	0.243	0.999	0.117
Very late recovery post-COVID-19 vs. controls	4.422	−51.384 to 60.229	0.498	0.333	0.601

COVID-19: coronavirus disease 2019; CI: confidence interval.

**Table 4:** The results of Egger’s and Begg’s tests for all comparisons between the studies analyzing carotid-femoral pulse wave velocity between COVID-19 participants at a different stage of the disease/recovery and controls.

Comparison	Egger’s Test			Begg’s Test	
	Intercept	95% CI	*p*	Kendall’s Tau	*p*
Early recovery post-COVID-19 patients vs. controls	−6.278	−19.952 to 7.396	0.240	−0.400	0.327
Mid-term recovery post-COVID-19 vs. controls	5.682	0.044 to 11.320	0.049	0.357	0.216
Very late recovery post-COVID-19 vs. controls	2.641	−25.036 to 30.319	0.439	0.999	0.117

COVID-19: coronavirus disease 2019; CI: confidence interval.

## Data Availability

The study protocol is available on the PROSPERO (ref. CRD42025642888), https://www.crd.york.ac.uk/prospero/; accessed on 27 January 2025. All collected data were presented in the publication and Supplementary Materials.

## Supplementary Materials

The following supporting information can be downloaded at https://www.mdpi.com/article/10.3390/life15040520/s1. Figure S1: PRISMA systematic review flow diagram; Figure S2: Forest plots for relations in brachial flow-mediated dilation between COVID-19 participants at a different stage of the disease/recovery; Figure S3: Forest plots for relations in carotid-femoral pulse wave velocity between COVID-19 participants at a different stage of the disease/recovery; Table S1: Reporting checklist for systematic review based on the PRISMA guidelines. Table S2: Clinical data of cohorts included in studies comparing post-COVID-19 participants and controls; Table S3: Clinical data of cohorts in studies assessing changes in selected parameters during follow-up; Table S4: Study design and main findings in studies comparing post-COVID-19 participants and non-COVID-19 controls; Table S5: Study design and main findings in studies assessing changes in selected parameters during follow-up.
