# Supplementary material for: Variability in Arterial Stiffness and Vascular Endothelial Function After COVID-19 During 1.5 Years of Follow-Up—Systematic Review and Meta-Analysis
Source: Life (Basel). 2025 Mar 21;15(4):520. doi: 10.3390/life15040520 (PMC12028431; doi:10.3390/life15040520)
Supplement: Supplementary file 1 [file life-15-00520-s001.zip › Life, supp. figures with approved changes.pdf]

Supplemental online material

Systematic Review: **Variability in arterial stiffness and vascular endothelial function after COVID-19 during 1.5 years of follow-up - systematic review and meta-analysis**

Danuta Loboda, Krzysztof S. Golba, Piotr Gurowiec, Aelita Bredelytė, Artūras Razbadauskas, and Beata Sarecka-Hujar

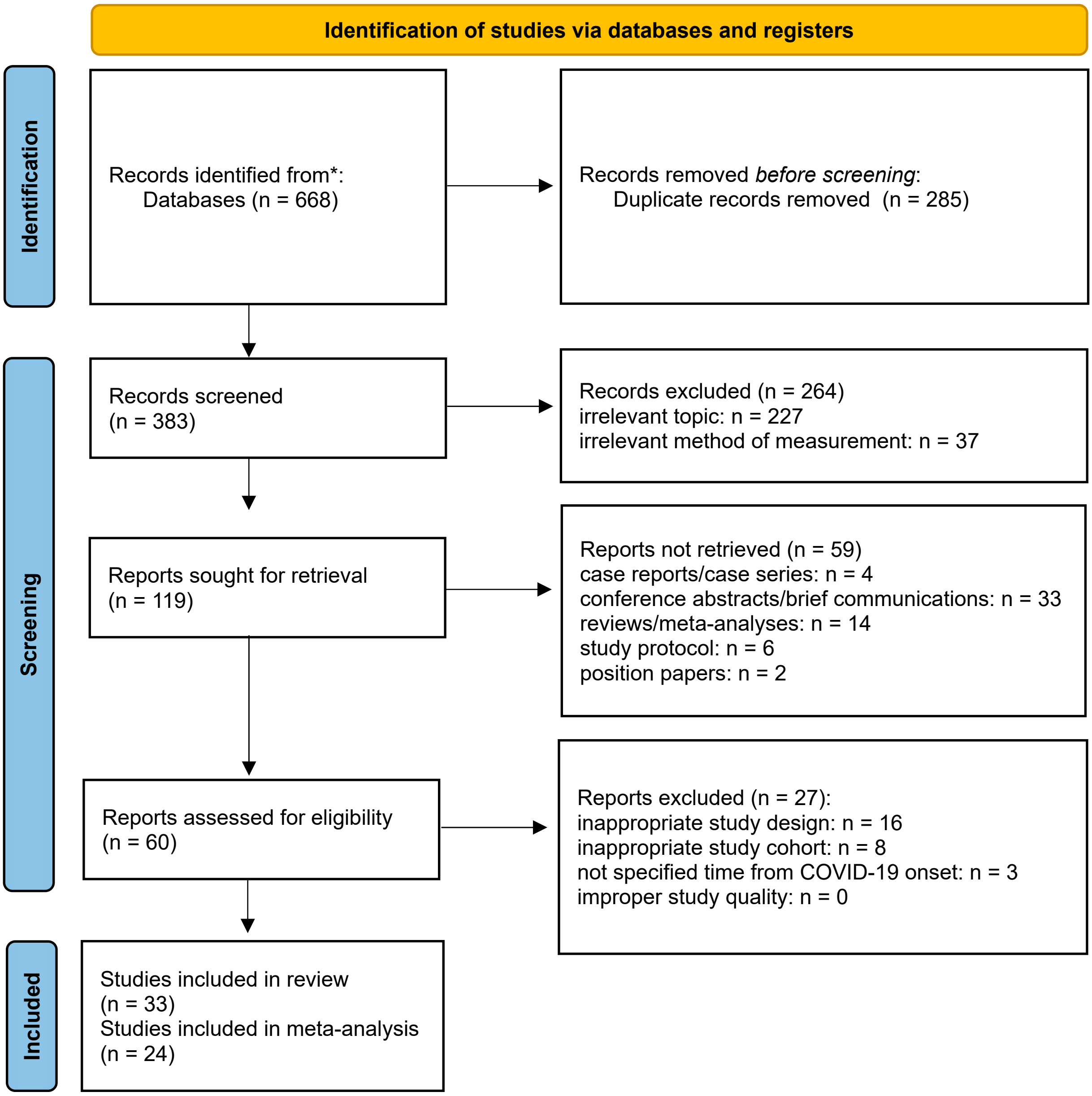

Supplementary Figure S1. PRISMA 2020 flow diagram.

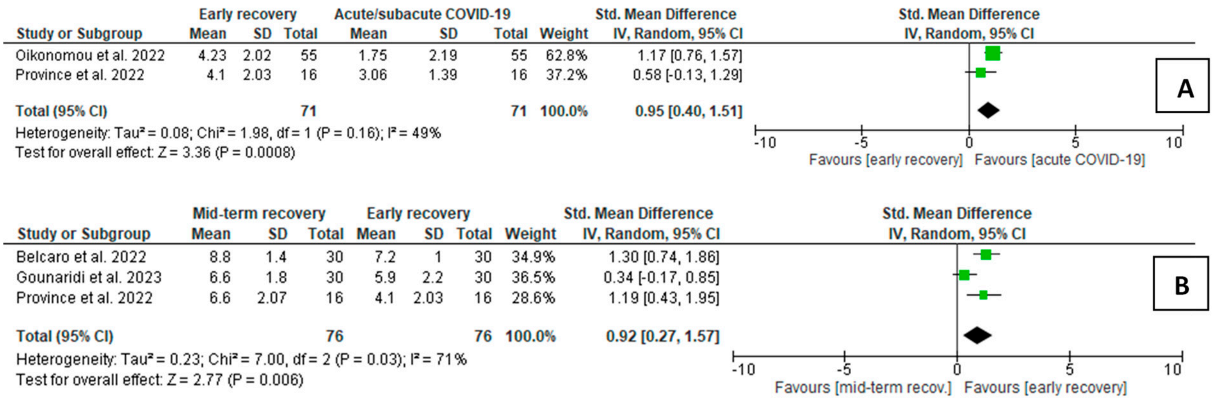

Supplementary Figure S2. Forest plots for relations in brachial flow-mediated dilation between COVID-19 participants at a different stage of the disease/recovery: A) early recovery COVID-19 patients vs acute/subacute COVID-19 patients; B) mid-term recovery COVID-19 patients vs early recovery post-COVID-19 patients; SD: standard deviation, CI: confidence interval; I^2: heterogeneity; df: degrees of freedom.

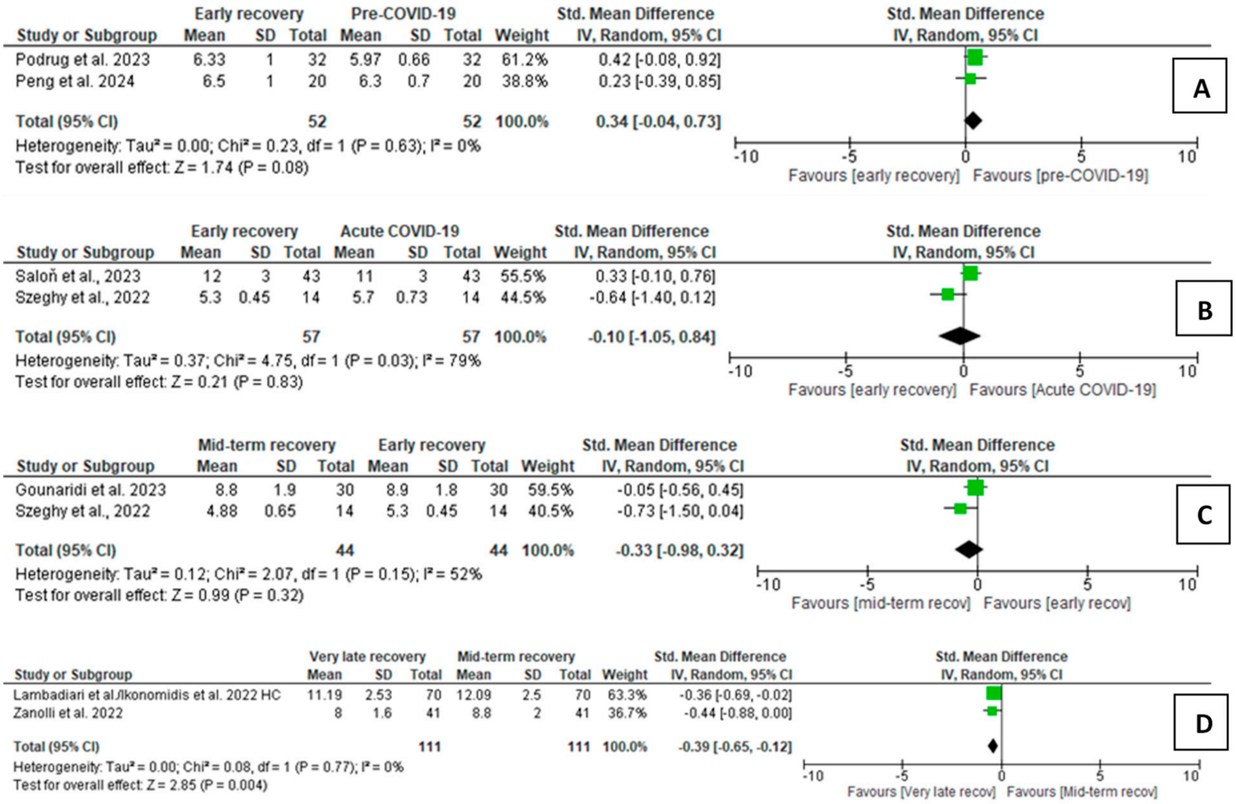

Supplementary Figure S3. Forest plots for relations in carotid-femoral pulse wave velocity between COVID-19 participants at a different stage of the disease/recovery: A) early recovery COVID-19 patients vs pre-COVID-19 patients; B) early recovery COVID-19 patients vs acute/subacute COVID-19 patients; C) mid-term recovery COVID-19 patients vs early recovery post-COVID-19 patients; D) very late recovery COVID-19 patients vs mid-term recovery COVID-19 patients. SD: standard deviation, CI: confidence interval; I^2: heterogeneity; df: degrees of freedom.
