# Supplementary material for: Variability in Arterial Stiffness and Vascular Endothelial Function After COVID-19 During 1.5 Years of Follow-Up—Systematic Review and Meta-Analysis
Source: Life (Basel). 2025 Mar 21;15(4):520. doi: 10.3390/life15040520 (PMC12028431; doi:10.3390/life15040520)
Supplement: Supplementary file 1 [file life-15-00520-s001.zip › Life, supp. figures.pdf]

Supplemental online material

Systematic Review: **Variability in arterial stiffness and vascular endothelial function after COVID-19 during 1.5 years of follow-up - systematic review and meta-analysis**

Danuta Loboda, Krzysztof S. Golba, Piotr Gurowiec, Aelita Bredelytė, Artūras Razbadauskas, and Beata Sarecka-Hujar

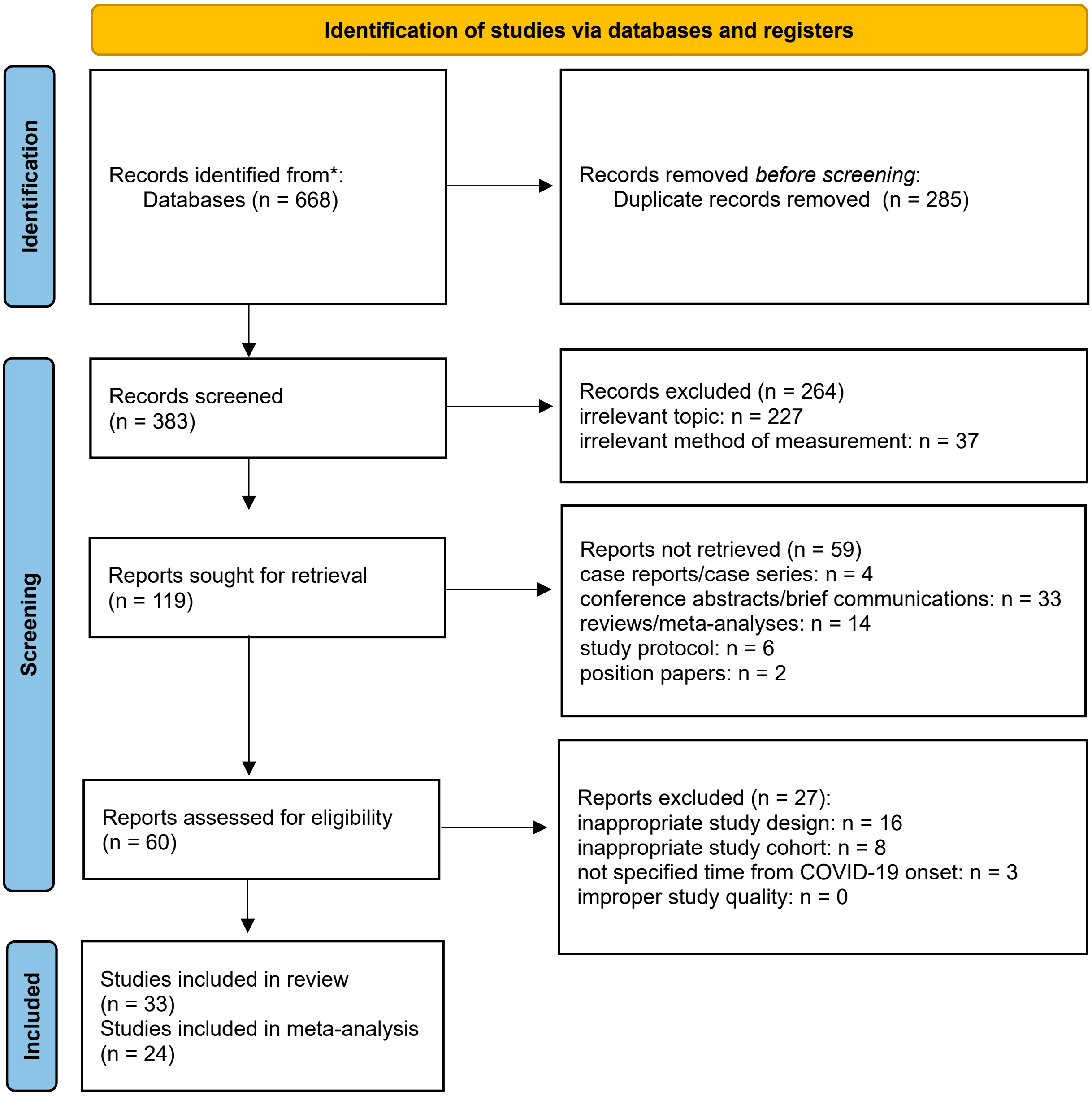

Supplementary Figure S1. PRISMA 2020 flow diagram.

	Early recovery			Acute/subacute COVID-19				Std. Mean Difference
Study or Subgroup	Mean	SD	Total	Mean	SD	Total	Weight	IV, Random, 95% CI
Oikonomou et al. 2022	4.23	2.02	55	1.75	2.19	55	62.8%	1.17 [0.76, 1.57]
Province et al. 2022	4.1	2.03	16	3.06	1.39	16	37.2%	0.58 [-0.13, 1.29]
**Total (95% CI)**			**71**			**71**	**100.0%**	**0.95 [0.40, 1.51]**

Heterogeneity: Tau² = 0.08; Chi² = 1.98, df = 1 (P = 0.16); I² = 49%
Test for overall effect: Z = 3.36 (P = 0.0008)

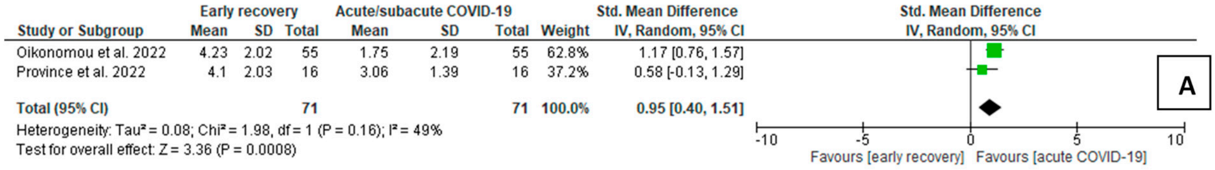

	Mid-term recovery			Early recovery				Std. Mean Difference
Study or Subgroup	Mean	SD	Total	Mean	SD	Total	Weight	IV, Random, 95% CI
Belcaro et al. 2022	8.8	1.4	30	7.2	1	30	34.9%	1.30 [0.74, 1.86]
Gounaridi et al. 2023	6.6	1.8	30	5.9	2.2	30	36.5%	0.34 [-0.17, 0.85]
Province et al. 2022	6.6	2.07	16	4.1	2.03	16	28.6%	1.19 [0.43, 1.95]
**Total (95% CI)**			**76**			**76**	**100.0%**	**0.92 [0.27, 1.57]**

Heterogeneity: Tau² = 0.23; Chi² = 7.00, df = 2 (P = 0.03); I² = 71%
Test for overall effect: Z = 2.77 (P = 0.006)

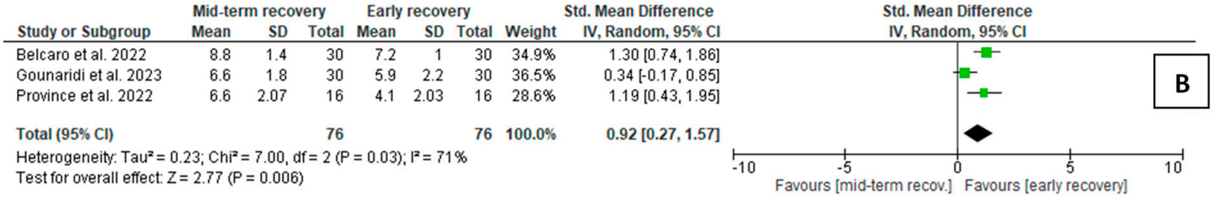

Supplementary Figure S2. Forest plots for relations in brachial flow-mediated dilation between COVID-19 participants at a different stage of the disease/recovery: A) early recovery COVID-19 patients vs acute/subacute COVID-19 patients; B) mid-term recovery COVID-19 patients vs early recovery post-COVID-19 patients; SD: standard deviation, CI: confidence interval; I²: heterogeneity; df: degrees of freedom.

	Early recovery			Pre-COVID-19				Std. Mean Difference
Study or Subgroup	Mean	SD	Total	Mean	SD	Total	Weight	IV, Random, 95% CI
Podrug et al. 2023	6.33	1	32	5.97	0.66	32	61.2%	0.42 [-0.08, 0.92]
Peng et al. 2024	6.5	1	20	6.3	0.7	20	38.8%	0.23 [-0.39, 0.85]
**Total (95% CI)**			**52**			**52**	**100.0%**	**0.34 [-0.04, 0.73]**

Heterogeneity: Tau² = 0.00; Chi² = 0.23, df = 1 (P = 0.63); I² = 0%
Test for overall effect: Z = 1.74 (P = 0.08)

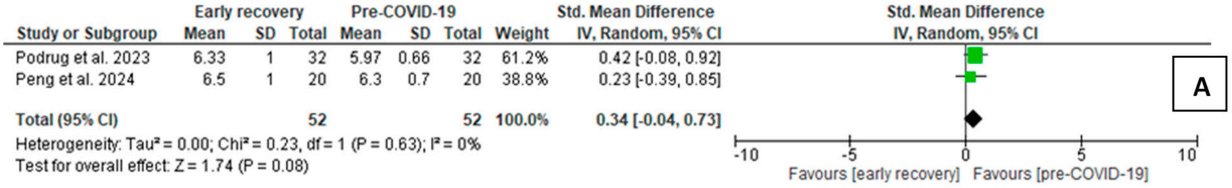

	Early recovery			Acute COVID-19				Std. Mean Difference
Study or Subgroup	Mean	SD	Total	Mean	SD	Total	Weight	IV, Random, 95% CI
Saloň et al., 2023	12	3	43	11	3	43	55.5%	0.33 [-0.10, 0.76]
Szeghy et al., 2022	5.3	0.45	14	5.7	0.73	14	44.5%	-0.64 [-1.40, 0.12]
**Total (95% CI)**			**57**			**57**	**100.0%**	**-0.10 [-1.05, 0.84]**

Heterogeneity: Tau² = 0.37; Chi² = 4.75, df = 1 (P = 0.03); I² = 79%
Test for overall effect: Z = 0.21 (P = 0.83)

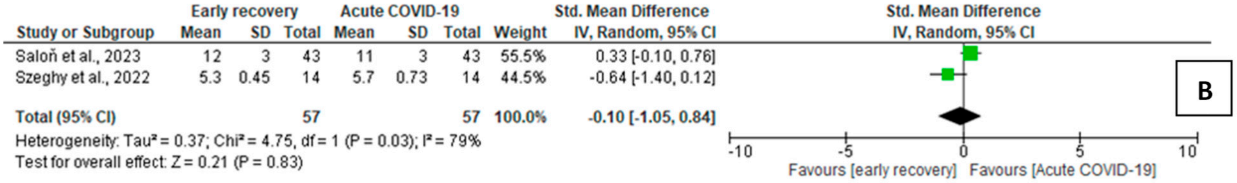

	Mid-term recovery			Early recovery				Std. Mean Difference
Study or Subgroup	Mean	SD	Total	Mean	SD	Total	Weight	IV, Random, 95% CI
Gounaridi et al. 2023	8.8	1.9	30	8.9	1.8	30	59.5%	-0.05 [-0.56, 0.45]
Szeghy et al., 2022	4.88	0.65	14	5.3	0.45	14	40.5%	-0.73 [-1.50, 0.04]
**Total (95% CI)**			**44**			**44**	**100.0%**	**-0.33 [-0.98, 0.32]**

Heterogeneity: Tau² = 0.12; Chi² = 2.07, df = 1 (P = 0.15); I² = 52%
Test for overall effect: Z = 0.99 (P = 0.32)

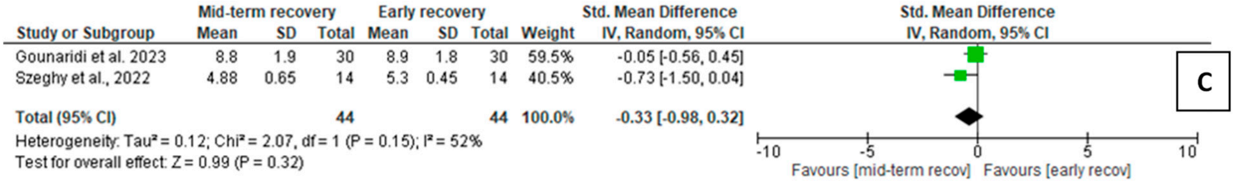

	Very late recovery			Mid-term recovery				Std. Mean Difference
Study or Subgroup	Mean	SD	Total	Mean	SD	Total	Weight	IV, Random, 95% CI
Lambadiari et al./Ikonomidis et al. 2022 HC	11.19	2.53	70	12.09	2.5	70	63.3%	-0.36 [-0.69, -0.02]
Zanolli et al. 2022	8	1.6	41	8.8	2	41	36.7%	-0.44 [-0.88, 0.00]
**Total (95% CI)**			**111**			**111**	**100.0%**	**-0.39 [-0.65, -0.12]**

Heterogeneity: Tau² = 0.00; Chi² = 0.08, df = 1 (P = 0.77); I² = 0%
Test for overall effect: Z = 2.85 (P = 0.004)

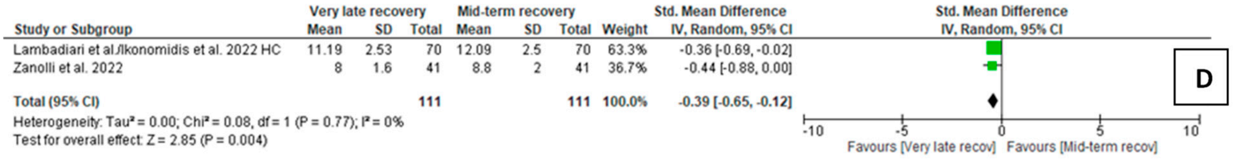

Supplementary Figure S3. Forest plots for relations in carotid-femoral pulse wave velocity between COVID-19 participants at a different stage of the disease/recovery: A) early recovery COVID-19 patients vs pre-COVID-19 patients; B) early recovery COVID-19 patients vs acute/subacute COVID-19 patients; C) mid-term recovery COVID-19 patients vs early recovery post-COVID-19 patients; D) very late recovery COVID-19 patients vs mid-term recovery COVID-19 patients. SD: standard deviation, CI: confidence interval; I²: heterogeneity; df: degrees of freedom.
