# Supplementary material for: Variability in Arterial Stiffness and Vascular Endothelial Function After COVID-19 During 1.5 Years of Follow-Up—Systematic Review and Meta-Analysis
Source: Life (Basel). 2025 Mar 21;15(4):520. doi: 10.3390/life15040520 (PMC12028431; doi:10.3390/life15040520)
Supplement: Supplementary file 1 [file life-15-00520-s001.zip › Life, suppl. tables with approved changes.pdf]

Supplemental online material

Systematic Review: **Variability in arterial stiffness and vascular endothelial function after COVID-19 during 1.5 years of follow-up - systematic review and meta-analysis**

Danuta Loboda, Krzysztof S. Golba, Piotr Gurowiec, Aelita Bredelytė, Artūras Razbadauskas, and Beata Sarecka-Hujar

Supplementary Table S1. Reporting checklist for systematic review based on the PRISMA guidelines.

		Reporting Item	Page Number
**Title**		Variability in arterial stiffness and vascular endothelial function after COVID-19 during 1.5 years of follow-up – systematic review and meta-analysis	1
Title	#1	Identify the report as a systematic review	1
**Abstract**			
Abstract	#2	Report an abstract addressing each item in the PRISMA 2020 for Abstracts checklist	1
**Introduction**			
Background/rationale	#3	Describe the rationale for the review in the context of existing knowledge	2, 3
Objectives	#4	Provide an explicit statement of the objective(s) or question(s) the review addresses	3
**Methods**			
Eligibility criteria	#5	Specify the inclusion and exclusion criteria for the review and how studies were grouped for the syntheses	3, 4
Information sources	#6	Specify all databases, registers, websites, organisations, reference lists, and other sources searched or consulted to identify studies. Specify the date when each source was last searched or consulted	3, 4
Search strategy	#7	Present the full search strategies for all databases, registers, and websites, including any filters and limits used	4
Selection process	#8	Specify the methods used to decide whether a study met the inclusion criteria of the review, including how many reviewers screened each record and each report retrieved, whether they worked independently, and, if applicable, details of automation tools used in the process	4
Data collection process	#9	Specify the methods used to collect data from reports, including how many reviewers collected data from each report, whether they worked independently, any processes for obtaining or confirming data from study investigators, and, if applicable, details of automation tools used in the process	4
Data items	#10a	List and define all outcomes for which data were sought. Specify whether all results that were compatible with each outcome domain in each study were sought (for example, for all measures, time points, analyses), and, if not, the methods used to decide which results to collect	4

Data items	#10b	List and define all other variables for which data were sought (such as participant and intervention characteristics, funding sources). Describe any assumptions made about any missing or unclear information	4
Study risk of bias assessment	#11	Specify the methods used to assess risk of bias in the included studies, including details of the tool(s) used, how many reviewers assessed each study and whether they worked independently, and, if applicable, details of automation tools used in the process	4
Effect measures	#12	Specify for each outcome the effect measure(s) (such as risk ratio, mean difference) used in the synthesis or presentation of results	4, 5
Synthesis methods	#13a	Describe the processes used to decide which studies were eligible for each synthesis (such as tabulating the study intervention characteristics and comparing against the planned groups for each synthesis (item #5))	4
Synthesis methods	#13b	Describe any methods required to prepare the data for presentation or synthesis, such as handling of missing summary statistics or data conversions	4, 5
Synthesis methods	#13c	Describe any methods used to tabulate or visually display results of individual studies and syntheses	4, 5
Synthesis methods	#13d	Describe any methods used to synthesise results and provide a rationale for the choice(s). If meta-analysis was performed, describe the model(s), method(s) to identify the presence and extent of statistical heterogeneity, and software package(s) used	4, 5
Synthesis methods	#13e	Describe any methods used to explore possible causes of heterogeneity among study results (such as subgroup analysis, meta-regression)	4, 5
Synthesis methods	#13f	Describe any sensitivity analyses conducted to assess robustness of the synthesised results	4, 5
Reporting bias assessment	#14	Describe any methods used to assess risk of bias due to missing results in a synthesis (arising from reporting biases)	4, 5
Certainty assessment	#15	Describe any methods used to assess certainty (or confidence) in the body of evidence for an outcome	4, 5
**Results**			
Study selection	#16a	Describe the results of the search and selection process, from the number of records identified in the search to the number of studies included in the review, ideally using a flow diagram (http://www.prisma-statement.org/PRISMAStatement/FlowDiagram)	5<br>Supp. Figure S1
Study selection	#16b	Cite studies that might appear to meet the inclusion criteria, but which were excluded, and explain why they were excluded	5
Study characteristics	#17	Cite each included study and present its characteristics	5;<br>Table 1<br>Table 2<br>Supp. Table 2<br>Supp. Table 3<br>Supp. Table 4<br>Supp. Table 5
Risk of bias in studies	#18	Present assessments of risk of bias for each included study	4;<br>Supp. Table 4<br>Supp. Table 5

Results of individual studies	#19	For all outcomes, present for each study (a) summary statistics for each group (where appropriate) and (b) an effect estimate and its precision (such as confidence/credible interval), ideally using structured tables or plots	Supp. Table 4<br>Supp. Table 5
Results of syntheses	#20a	For each synthesis, briefly summarise the characteristics and risk of bias among contributing studies	14, 16, 17;<br>Table 3,<br>Table 4
Results of syntheses	#20b	Present results of all statistical syntheses conducted. If meta-analysis was done, present for each the summary estimate and its precision (such as confidence/credible interval) and measures of statistical heterogeneity. If comparing groups, describe the direction of the effect	8-12;<br>Figure 1<br>Figure 2
Results of syntheses	#20c	Present results of all investigations of possible causes of heterogeneity among study results	8-12
Results of syntheses	#20d	Present results of all sensitivity analyses conducted to assess the robustness of the synthesised results	8-12
Risk of reporting biases in syntheses	#21	Present assessments of risk of bias due to missing results (arising from reporting biases) for each synthesis assessed	8-12
Certainty of evidence	#22	Present assessments of certainty (or confidence) in the body of evidence for each outcome assessed	8-12
**Discussion**			
Results in context	#23a	Provide a general interpretation of the results in the context of other evidence	12-21
Limitations of included studies	#23b	Discuss any limitations of the evidence included in the review	20, 21
Limitations of the review methods	#23c	Discuss any limitations of the review processes used	20, 21
Implications	#23d	Discuss implications of the results for practice, policy, and future research	21
**Other information**			
Registration and protocol	#24a	Provide registration information for the review, including register name and registration number, or state that the review was not registered	1, 3
Registration and protocol	#24b	Indicate where the review protocol can be accessed, or state that a protocol was not prepared	21
Registration and protocol	#24c	Describe and explain any amendments to information provided at registration or in the protocol	21
Support	#25	Describe sources of financial or non-financial support for the review, and the role of the funders or sponsors in the review	21
Competing interests	#26	Declare any competing interests of review authors	21
Availability of data, code, and other materials	#27	Report which of the following are publicly available and where they can be found: template data collection forms; data extracted from included studies; data used for all analyses; analytic code; any other materials used in the review	21

Supplementary Table S2. Clinical data of cohorts included in studies comparing post-COVID-19 participants and controls.

Study	Compared study groups	Time since COVID-19 diagnosis	Acute COVID-19 severity	Post-COVID-19 symptoms	CV risk factors/ ASCVD

Ambrosino et al. [26], 2021	133 post-COV early recovery (non-CR)	16.7 (18.5) days from swab test negativization	70.7% HOSP (ICU)<br>30.8% severe COV<br>69.2% critical COV	-	History of CV events 14.3%,<br>AH 51.1%,<br>DM 15.8%,<br>Hypercholesterolemia 9.0%,<br>Obesity 27.1%,<br>Smoking 9.0%
	133 CON (CV)	-	-	-	Matched for age, sex, and major CV risk factors
Ciacci et al. [93], 2023	20 acute COV	Within 48h after hospital admission	HOSP,<br>with COVID-19 pneumonia	-	CV disease 20%,<br>AH 65%,<br>DM 25%,<br>Dyslipidemia 30%
	20 CON (CV)	-	-	-	Matched for age, sex, and CV risk factors
	20 CON (PN)	-	-	-	HOSP, with community acquired pneumonia
Dashoundhi et al. [92], 2023	18 acute COV	Acute COVID-19	Mild COV	-	Healthy adults
	18 CON (H)	-	-	-	Age and sex-matched apparently healthy participants
Ergül et al. [95], 2022	63 post-COV early recovery	2 months after hospital discharge	HOSP	-	-
	29 CON group	-	-	-	Age-and sex-matched
Faria et al. [105], 2023	19 post-COV mid-term recovery (LC)	89.00 (43.00) days after hospital discharge.<br>Hospital stay: 12.00 (7.00) days	HOSP (wards or ICU).<br>Lung involvement on chest CT: 0–25%: 3 (16%);<br>25–50%: 11 (58%);<br>50–75%: 5 (26%)	100% with LC<br>Symptom score (0-100): 20.25 (3.9)	Free of CV or kidney diseases,<br>AH 1 (5%),<br>DM 1 (5%)
	19 CON (H)	-	-	-	Healthy adults
Gao et al. [29], 2022	86 post-COV very late recovery (LC)	327 (318-337) days after diagnosis	78 (91%) HOSP<br>45 (52%) moderate COV<br>27 (31%) severe COV<br>14 (17%) critical COV	LC:<br>25 (29%) with exertional shortness of breath, 33 (38%) with chest discomfort	CAD 15%,<br>AH 37%,<br>DM 16%,<br>Hypercholesterolemia 19%
	28 CON (H)	-	-	-	Healthy, age- and sex-matched

	30 CON (CV)	-	-	-	Risk factors-matched
Gounaridi et al. [98], 2023	60 post-COV early recovery	1 month post-acute disease	15 (25.0%) mild COV, non-HOSP; 36 (60.0%) moderate COV, HOSP; 9 (15.0%) severe COV, HOSP	-	AH 19 (20.0%), DM 1 (1.7%), Dyslipidemia 19 (31.7%), Smoking 22 (37%)
	60 CON (CV)	-	-	-	Age-, sex- and CV risk factors matched
Ikonomidis et al. [12], 2022	70 post-COV very late recovery	12 months after diagnosis	24 (34.28%) mild COV, non-HOSP; 23 (32.85%) moderate COV, HOSP; 23 (32.85%) severe COV, HOSP	4.25% with LC	Healthy adults
	70 CON (H)	-	-	-	Healthy adults
Jud et al. [10], 2021	14 post-COV late recovery	28.6 (3.0) weeks post infection	14 (100.0%) with COVID-19 pneumonia 3 (21.4%) with ARDS	-	AH 6 (42.9%), DM 0 (0.0%), Hypercholesterolemia 6 (42.9%), Hypertriglyceridemia 2 (14.3%), smoking: current 0 (0.0%), ex-smokers 6 (42.9%), non-smokers 8 (57.1%), Family history of ASCVD 5 (35.7%)
	14 CON (H)	-	-	-	Sex-matched, healthy
	14 CON (CV)	-	-	-	Age and sex-matched, with ASCVD
Lambadiari et al. [11], 2021	70 Post-COV mid-term recovery (LC)	≥ 4 months after infection	24 (34.28%) mild COV, non-HOSP; 23 (32.85%) moderate COV, HOSP; 23 (32.85%) severe COV, HOSP	26 (37.87%) with LC	Healthy adults (except for untreated hypertension)
	70 CON (H)	-	-	-	Healthy adults
	70 CON (AH)(CV)	-	-	-	Hypertensive
Luck et al. [94], 2023	14 subacute COV	23 (9) since positive PCR test	-	Symptom score (0–18): 4 (3)	Healthy young adults, collegiate athletes

	10 CON (H)	-	-	-	Healthy young adults
Mansiroglu et al. [97], 2022	80 post-COV early recovery	35 days (range: 25-178) after diagnosis	Mild COV, non-HOSP	65% with at least one symptom	AH: 2 (2.25%),<br>DM: 2 (2.25%),<br>Dyslipidemia: 1 (1.3%),<br>Family history of CAD: 26 (32.5%),<br>Smoking: 19 (23.8%)
	81 CON (H)	-	-	-	Healthy
Mclaughlin et al. [108], 2023	17 post-COV very-late recovery (LC)	1.36 (0.51) years of illness duration	-	100% with LC	-
	17 CON (H)	-	-	-	Healthy, age-matched
	17 CON (ME/CFS)	-	-	-	With myalgic encephalomyelitis/chronic fatigue syndrome
Nandadeva et al. [27] 2021	8 post-COV mid-term recovery (N-LC)	12 (5) weeks from diagnosis	-	100% asymptomatic	Healthy young adults, nonsmokers
	8 post-COV mid-term recovery (LC)	14 (4) weeks from diagnosis	-	100% with LC. Symptom severity (1–10): 4 (1)	
	12 CON (H)	-	-	-	Healthy
Nandadeva et al. [107] 2023	12 post-COV very-late recovery (LC)	15 months (range 3–30) since diagnosis	-	100% with LC	AH 3 (2%),<br>DM 2 (1%),<br>Dyslipidemia 3 (2%),<br>Smoking 0 (0%)
	11 CON (H)	2 with prior asymptomatic COV	-	-	Age-, and BMI-matched CON without LC
Oikonomou et al., 2022 [28]	73 acute COV	24-72 h after hospital admission	HOSP, 46 (37%) ICU	-	CAD 7 (9.6%),<br>AH 34 (46.6%),<br>DM 20 (27.4%),<br>Dyslipidemia 23 (31.5%)
	73 CON (CV)	-	-	-	Propensity score matched
	55 post-COV early recovery	28 days after hospital discharge. Hospital stay 14 (8-26) days	HOSP	-	CAD 3 (5.5%),<br>AH 21 (38.2%),<br>DM 12 (21.8%),<br>Dyslipidemia 14 (25.5%)
	55 post-COV late recovery (LC)	6 months after hospital discharge		58% with LC	
	55 CON (CV)	-	-	-	Propensity score matched

Oikonomou et al., 2023 [99]	34 post-COV early recovery	1 month (28 days) after hospital discharge	-	56% symptomatic	CAD 8 (23.5%), AH 13 (38.2%), DM 5 (14.7%), Dyslipidemia 16 (47.1%), Smoking 19 (55.9%)
	30 post-COV late recovery (LC)	6 months after hospital discharge	-	30% with LC	
	34 CON (CV)	-	-	-	Age-, sex- and CV risk factors propensity score matched
Oliveira et al. [90], 2021	98 acute COV	First 24–48h of hospitalization	HOSP, 18 (18%) ICU	-	AH 52 (63%), DM 29 (30%)
	82 CON (PN)	-		-	Respiratory symptoms with negative COV RT-PCR
Province et al., 2022 [91]	16 subacute COV	1M: 25 (5) day following positive SARS-CoV-2 test	-	Symptom severity score (0-100): 1M: 4 (7)	Healthy young adults
	16 post-COV early recovery	2M: 57 (7) day, 3M: 87 (8) day following positive SARS-CoV-2 test		Symptom severity score (0-100): 2M: 2 (6), 3M: 3 (8)	
	12 post-COV mid-term recovery (N-LC)	4M: 119 (13) day 6M: 174 (15) day following positive SARS-CoV-2 test		Symptom severity score (0-100): 4M: 1 (4), 6M: 0 (0)	
	20 CON (H)	-	-	-	Healthy young adults
Ratchford et al, [89] 2021	11 subacute COV	25 (5) days since symptom onset (n = 10); 24 (6) days after positive testing (n = 11)	Mild, lingering symptoms	Symptom severity score (0–100): 15.0 (12.2)	Healthy young adults, nonsmokers
	20 CON (H)	-	-	-	Healthy young adults
Riou et al. [103], 2021	27 post-COV mid-term recovery	3 months after hospitalization. Hospital stay: 14 (8–42) days	HOSP, mild-to-moderate COV (n = 16), severe-to-critical COV (n = 11), ICU for ARDS (n = 11)	-	AH 13 (48%), DM 7 (26%), Sleep apnea syndrome 6 (22%), Former or active smokers 6 (22%)
	9 CON (CV)	-	-	-	Age- and sex- matched

Schnaubelt et al., [33] 2021	22 acute COV	During hospitalization	Acutely ill with cardiorespiratory symptoms, HOSP	-	CAD 8 (36%),<br>Cerebrovascular disease 2 (9%),<br>PAD 2 (9%),<br>AH 15 (68%),<br>DM 7 (32%),<br>Hyperlipidaemia 6 (27%),<br>Smoker 8 (36%),<br>CKD 5 (23%)
	22 CON (PN) (CV)	-	-	-	Age- and sex-matched, acutely ill hospitalized adults with cardiorespiratory symptoms
Skow et al. [96], 2022	23 post-COV early recovery	30 (9) days post diagnosis	Mild to moderate COV (Omicron variant), non-HOSP	4 (17.4%) symptomatic. Symptom severity (0-10): 3 (1)	Healthy young adults, nonsmokers
	13 CON (H)	-	-	-	Vaccinated healthy young adults
Tudoran et al. [100], 2023	54 Post-COV early recovery (N-MS)	63 (56–70) days since diagnosis	Mild/moderate COV	100% symptomatic. PCFS (0-4): 1 (1–2)	Healthy premenopausal women, non-smokers
	67 Post-COV early recovery (MS)	56 (56–70) days since diagnosis		100% symptomatic. PCFS (0-4): 2 (1–2)	Premenopausal women with MS, non-smokers
	40 CON (H)	-	-	-	Age-matched, healthy
van der Sluijs et al [104], 2023	31 post-COV mid-term recovery (LC)	175 (126–235) days post infection (for the whole group)	Mild COV, non-HOSP	100% with LC	MI 3 (10%),<br>Stroke 1 (3%),<br>AH 3 (10%),<br>DM 2 (7%),<br>Hypercholesterolemia 7 (23%),<br>Smoking: current 1 (3%), former 13 (42%), never 17 (55%)
	31 CON (CV)	-	-	-	Age- and sex-matched
	97 post-COV mid-term recovery (LC)	175 (126–235) days post infection	Mild COV, non-HOSP	32% with LC	MI 4 (4%),<br>Stroke 2 (2%),<br>AH 17 (18%),<br>DM 4 (4%),<br>Hypercholesterolemia 14 (15%),

					Smoking: current 6 (6%), former 32 (33%), never 58 (60%)
	49 CON (CV)	-	-	-	Age- and sex-matched
Vidya et al. [106], 2023	IA: 32 post-COV mid-term recovery with DM	≥ 4 months post infection	Mild and moderate COV	-	DM 100%, non-smokers
	IB: 28 CON with DM (CV)	-		-	DM
	IIA: 20 post-COV mid-term recovery with AH	≥ 4 months post infection	Mild and moderate COV	-	AH 100%, non-smokers
	IIB: 20 CON with AH (CV)	-		-	AH
	IIIA: 25 post-COV mid-term recovery with obesity	≥ 4 months post infection	Mild and moderate COV	-	Healthy with obesity (BMI >30 kg/m^2), non-smokers
	IIIB: 25 CON with obesity (CV)			-	Healthy, with obesity
Zanoli et al. 2022 [35] (Study 1)	45 post-COV mid-term recovery (LC)	18 (3) weeks since COV onset	HOSP, 17% moderate COV (no O_2 therapy), 49% moderate COV (low-flux O_2), 34% severe COV (high flux O_2/CPAP)	63% with LC	Healthy adults
	45 post-COV very-late recovery	33 (5) weeks since COV onset		-	
	180 CON (H)	-	-	-	Age-, sex-, and BMI-matched

(CV) control group: participants with cardiovascular risk factors or atherosclerotic cardiovascular diseases; (H) control group: healthy adults; (LC) group: ≥ 30% of participants in group with long-COVID-19 syndrome; (ME/CFS) control group: participants with myalgic encephalomyelitis/chronic fatigue syndrome; (MS) group: participants with metabolic syndrome; (N-LC) group: asymptomatic participants; (N-MS) group: participants without metabolic syndrome; (PN) control group: participants with non-COVID-19 pneumonia or respiratory symptoms;

ARDS: acute respiratory distress syndrome; AH: arterial hypertension; ASCVD: atherosclerotic cardiovascular disease; BMI: body mass index; CAD: coronary artery disease; CON: control group; COV: COVID-19 group; CPAP: continuous positive airway pressure; CT: computed tomography; CV: cardiovascular; DM: diabetes; HOSP: hospitalized; ICU: intensive care unit; LC: long-COVID-19 syndrome; MI: myocardial

infarction; MS: metabolic syndrome; O_2: oxygen therapy; PAD: peripheral arterial disease; PCFS: post-COVID-19 functional scale; PCR: polymerase chain reaction test; SARS-CoV-2: Severe acute respiratory syndrome coronavirus 2

Supplementary Table S3. Clinical data of cohorts in studies assessing changes in selected parameters during follow-up.

Study	Study group in F/U	Time since diagnosis	Acute phase severity	Post-COVID symptoms	ASCVD risk factors
Belcaro et al. [111], 2022	30 Post COV in early recovery period (non-Pycnogenol® group)	≥ 2 months<br>≥ 3 months<br>after diagnosis	Symptomatic	QoL mood and fatigue Score (0-35):<br>≥2M: 23.4 (1.2 1);<br>≥3M: 27.3 (1.7 3)	Healthy adults
	30 Post-COV in mid-term recovery period (non-Pycnogenol® group)	≥ 5 months after diagnosis		QoL mood and fatigue Score [0-35]:<br>≥5M: 26.7 (1.5)	
Gounaridi et al. [98], 2023	30 Post-COV in early recovery period (non-CR group)	1 month post-acute disease	10 (33.3%) mild COV, non-HOSP<br>18 (60.0%) moderate COV, HOSP<br>2 (6.7%) severe COV, HOSP	-	AH 5 (16.7%),<br>DM 0 (0.0%),<br>Dyslipidemia 10 (33.3%),<br>Smoking, 11 (37.0%)
	30 Post-COV in mid-term recovery period (non-CR group)	4 months post-acute disease			
Lambadiari et al. [11], 2021	70 Post-COV in mid-term recovery period	4 months after diagnosis	24 (34.28%) mild COV, non-HOSP<br>23 (32.85%) moderate COV, HOSP<br>23 (32.85%) severe COV, HOSP	37.87% with LC	Healthy adults (except for untreated hypertension)
Ikonomidis et al. [12], 2022	70 Post-COV in very late recovery period	12 months after diagnosis		4.25% with LC	
Oikonomou et al. [28], 2022	55 in acute COV period	24-72h after hospital admission	-	-	CAD 3 (5.5%),<br>AH 21 (38.2%),<br>DM 12 (21.8%),<br>Dyslipidemia 14 (25.5%)
	55 Post-COV in early recovery period	1-month (28 days) after hospital discharge.<br>Hospital stay: 14 (8- 26) days	-	-	
	55 Post-COV in late recovery period	6 months after hospital discharge	-	58% with LC	
Oikonomou et al. [99],	34 Post-COV in early recovery period	1 month (28 days) after hospital discharge	-	56% with LC	CAD 8 (23.5%),<br>AH 13 (38.2%),

2023	34 Post-COV in late recovery period	6 months after hospital discharge		30% with LC	DM 5 (14.7%),<br>Dyslipidemia 16 (47.1%),<br>Smoking 19 (55.9%)
Peng et al. [102], 2024	37 in pre-COV period	Before COV	-	-	Healthy young adults
	20 post-COV in early recovery period	<3.8 months since diagnosis	Mild COV, non-HOSP	-	
	17 post-COV in mid-term recovery period	>3.8 months since diagnosis			
Podrug et al. [101], 2023	32 in pre-COV period	Up to 2 yrs before COV	-	-	AH 3 (9%),<br>DM 2 (6%),<br>Dyslipidemia 0 (0%),<br>Familial history of CV disease 7 (22%),<br>Smoking: no 17 (53%), yes 7 (22%), ex-smoker 8 25%)
	32 post-COV in early recovery period	73 (10) days since COV onset	Non-HOSP	Asymptomatic	
Province et al. [91], 2022	16 in subacute COV period	1M: 25 (5) day following positive SARS-CoV-2 test	-	Symptom severity score (0-100): 1M: 4 (7)	Healthy young adults
	16 Post-COV in early recovery period	2M: 57 (7) day, 3M: 87 (8) day following positive SARS-CoV-2 test		Symptom severity score (0-100): 2M: 2 (6), 3M: 3 (8)	
	12 Post-COV in mid-term recovery period	4M: 119 (13) day, 6M: 174 (15) day following positive SARS-CoV-2 test		Symptom severity score (0-100): 4M: 1 (4), 6M: 0 (0)	
Saloň et al. [14], 2023	35 in subacute COV period	At hospital discharge (if a negative PCR test) or on the 10th day after hospital discharge (if a positive PCR test).<br>Hospital stay: 7.0 (4.9) days	HOSP,<br>2 (5.7%) at ICU	25 patients with dyspnea	-
	35 post-COV in early recovery period	60 days after hospital discharge		-	
Szeghy et al. [110], 2022	14 in subacute COV period	1M: 25 (6) day following positive SARS-CoV-2 test	-	Symptom severity score (0-100): 1M: 3.32 (3.10)	Healthy young adults
	14 post-COV in early recovery period	2M: 57 (7) day, 3M: 87 (8) day following positive SARS-CoV-2 test		Symptom severity score (0-100): 2M: 1.36 (1.26), 3M: 1.18 (1.73)	
	12 post-COV in mid-term recovery period	6M: 174 (15) day following positive SARS-CoV-2 test		Symptom severity score (0-100): 4M: 0.81 (1.14), 6M: 0.03 (0.07)	

Teixeira DO Amaral et al. [112], 2022	20 post-COV in early recovery period (non-CR group)	35.7 (5.1) days after hospital discharge. Hospital stay: 7 (5) days	HOSP, 2 (10%) ICU	85% symptomatic at baseline and in F/U (LC). The quantity of symptoms: at baseline: 2.6 (1.5) vs. F/U: 2.3 (1.9), p = 0.464	CV disease 2 (10%), AH 11 (55%), DM 1 (5%), Dyslipidemia 2 (10%), Obesity 13 (65%), Smoking: never 15, current 1, former 4 participants
	20 post-COV in mid-term recovery period (non-CR group)	After 4 months (12 weeks) of F/U			
Zanoli et al. [35] (Study 2), 2022	41 post-COV in mid-term recovery period	21 (5) weeks after COV onset	HOSP, 25% moderate COV (no O_2 therapy), 44% moderate COV (low-flux O_2), 31% severe COV (high flux O_2/CPAP)	69% with LC	Healthy adults
	41 post-COV in very-late recovery period	48 (6) weeks after COV onset (after 27 weeks of F/U)		58% with LC	

(non-CR group): patients non participated in cardiopulmonary rehabilitation; (non-Pycnogenol® group): patients not receiving the supplement;

AH: arterial hypertension; CAD: coronary artery disease; CON: control group; COV: COVID-19 group; CPAP: continuous positive airway pressure; CV: cardiovascular; DM: diabetes; F/U: follow up; HOSP: hospitalized; ICU: intensive care unit; LC: long-COVID-19 syndrome; O_2: oxygen therapy; QoL: quality of life; PCR: polymerase chain reaction test; SARS-CoV-2: Severe acute respiratory syndrome coronavirus 2

Supplementary Table S4. Study design and main findings in studies comparing post-COVID-19 participants and non-COVID-19 controls.

Study	Design	Assessment	Main findings	Quality (NOS)
Ambrosino et al. [26], 2021	Observational case-control	FMD, Vivid E95 ultrasound (GE Healthcare, Illinois, USA)	In early recovery, FMD was lower in post-COV: 3.2 (2.6)% vs. CON: 6.4 (4.1)%, p < 0.001. The recent COV was an independent predictor of FMD values (β = -0.427, p < 0.001). FDM directly correlated with the severity of pulmonary impairment, i.e., arterial oxygen tension (rho = 0.247, p = 0.004), forced expiratory volume in 1 s (rho = 0.436, p < 0.001), forced vital capacity (rho = 0.406, p < 0.001), and diffusing capacity for carbon monoxide (rho = 0.280, p = 0.008).	8
Ciacci et al. [93], 2023	Observational cross-sectional	FMD, Samsung HS30 (Samsung, Seoul, Korea)	In acute COV, FMD was lower in the COV group: 2.1 (0–5.7) vs. the healthy CON: 5.6 (4.7–7.4), p < 0.001. No difference between the acute-COV group and participants with non-COV pneumonia: 2.4 (1.3–5.7), p = 0.22.	8

Dashoundhi et al. [92], 2023	Observational cross-sectional	cfPWV, Periscope (Genesis Medical Systems, India)	In acute COV, cfPWV was higher in the COV group: 8.84 (1.95) vs. the healthy CON: 6.11 (1.54), $p < 0.01$.	7
Ergül et al. [95], 2022	Observational cross-sectional	FMD, unspecified ultrasound machine with 5- to 13-MHz linear transducer	In early recovery, FMD was lower in the post-COV: 17.4 (12.6–24.3) vs. CON: 28.8 (21.1–42.9), $p < 0.001$.<br>COV (OR 3.611, 95% CI 1.069–12.198, $p = 0.039$) and BMI (OR 1.122, 95% CI 1.023–1.231, $p = 0.015$) were independent predictors of endothelial dysfunction.	7
Faria et al. [105], 2023	Observational cross-sectional	FMD, Vivid E9 ultrasound (GE, Horten, Norway)<br>cfPWV, ComplioR device (Artech Medical)	In mid-term recovery:<br>FMD was 45% lower in the post-COV: 4.52 vs. the healthy CON: 8.42, $p = 0.001$ (raw FMD data not available; parameters calculated based on the mean change in absolute brachial artery diameter)<br>cfPWV was 16% higher in the post-COV: 8.6 (0.2) vs. the healthy CON: 7.4 (0.2), $p = 0.04$.	8
Gao et al. [29], 2022	Observational cross-sectional	FMD, Vivid E95 ultrasound (GE Medical System, Horten, Norway)	In very-late recovery, FMD was lower in the post-COV: 3.5 (2.2-4.6)% vs. the healthy CON: 7.7 (5.1-10.7)%, $p < 0.001$, and the risk-matched CON: 6.9 (5.5-9.4)%, $p < 0.001$.<br>No differences in FMD existed among groups with different COV severity: moderate: 3.8 (2.2–5.4)%, severe: 3.3 (2.3–4.4)%, critical: 3.2 (0.5–4.1)%, $p = 0.262$.	8
Gounaridi et al. [98], 2023	Prospective randomized control	FMD, Vivid Ultrasound (GE, Milwaukee, WI, US)<br>cfPWV, SphygmoCor device (AtCor Medical, Sydney, NSW, Australia)	In early recovery:<br>FMD was reduced in the post-COV: 6.1 (1.9)% vs. CON: 7.4 (3.2)%, $p = 0.02$.<br>cfPWV was increased in the post-COV: 8.4 (1.6) m/s vs. CON: 7.3 (0.8) m/s, $p < 0.001$.	7
Ikonomidis et al. [12], 2022	Observational case-control	FMD, Vivid E95 ultrasound (GE Medical Systems, Horten, Norway)<br>cfPWV, Complior system (Alam Medical, Vincennes, France)	In very-late recovery, FMD and cfPWV remained impaired in the post-COV vs. CON:<br>FMD: 6.49 (2.25)% vs. 9.06 (2.11)%, $p < 0.001$<br>cfPWV: 11.19 (2.53) m/s vs. 10.04 (1.80) m/s, $p = 0.05$	8
Jud et al. [10], 2021	Observational cross-sectional	FMD, Siemens ACUSON S2000™ ultrasound (Siemens Healthcare Corp., Erlangen, Germany)<br>cfPWV, Mobil-O-Graph® device (I.E.M., Aachen, Germany)	In late recovery:<br>FMD was similar within all three groups; the post-COV: 4.44 (2.90)% vs. the healthy CON: 4.58 (3.48)% vs. ASCVD CON: 3.17 (2.95)%, $p \geq 0.05$.<br>cfPWV was higher in the post-COV: 10.75 (8.10–11.45) m/s vs. the healthy CON: 5.70 (5.38–6.05) m/s, $p < 0.001$, but similar in the post-COV and ASCVD CON: 9.95 (8.40–11.60) m/s, $p \geq 0.05$.	6
Lambadiari et al. [11], 2022	Observational case-control	FMD, Vivid E95 (GE Medical Systems, Horten, Norway)<br>cfPWV, Complior system (Alam Medical, Vincennes, France)	In mid-term recovery:<br>FMD was lower in the post-COV: 5.86 (2.82)% ($p = 0.002$) and hypertensive CON: 5.80 (2.07)% ($p = 0.002$) than in the healthy CON: 9.06 (2.11%), but similar between the post-COV and hypertensive CON, $p = 0.872$.	8

			FMD was lower in the symptomatic (LC) subgroup: 5.99 (2.43)% vs. 4.99 (5.14)%, p = 0.036.<br>cfPWV was higher in the post-COV: 12.09 (2.50) m/s (p = 0.036) and hypertensive CON: 11.92 (2.94) m/s (p = 0.045) compared to the healthy CON: 10.04 (1.80) m/s.<br>cfPWV was higher in the symptomatic (LC) subgroup: 12.27 (2.95) m/s vs. 11.28 (3.11) m/s, p = 0.032.<br>FMD or cfPWV did not vary with COVID-19 severity (p values not given).	
Luck et al. [94], 2023	Observational cross-sectional	FMD, Logiq eR7 ultrasound, and L4-12T-RS transducer (GE Medical Systems, Milwaukee, WI)	In early recovery, FMD was decreased in the post-COV: 6.50 (2.06)% vs. CON: 11.07 (4.09)%, p < 0.01, but did not differ when allometrically scaled to account for differences in baseline diameter, p = 0.058.	6
Mansiroglu et al. [97], 2022	Observa-tional case-control	FMD, Vivid S6 with 4 MHz transducer (GE Vingmed Ultrasound AS, Horten, Norway)	In early recovery, FMD was lower in the post-COV: 9.52 (5.98) vs. the healthy CON: 12.01 (6.18), p = 0.01.	7
Mclaughlin et al. [108], 2023	Observational case-control	FMD, unspecified ultrasound device with 12 MHz linear transducer (Siemens, Erlangen, Germany)	In very-late recovery, FMD was lower in the post-COV group: 6.99 (4.33)% (p = 0.022) and ME/CFS CON group: 6.60 (3.48)% (p = 0.019) compared to the healthy CON group: 11.30 (4.44)%.<br>There was no difference in FDM between the post-COV and ME/CFS groups, p = 0.949.	6
Nandadeva et al. [27], 2021	Observational cross-sectional	FMD, Logiq P5 ultrasound with 11-MHz linear transducer (GE, Milwaukee, WI)<br>cfPWV, SphygmoCor, (Atcor XCEL 1.3 software, Sydney, Australia)	In mid-term recovery, FMD (p = 0.097) and cfPWV (p = 0.173) were similar in the whole post-COV group compared to CON.<br>FMD was lower in the LC: 3.8 (0.6)% vs. the non-LC: 6.8 (0.9)%, p = 0.007, and the healthy CON: 6.8 (0.6)%, p = 0.003, but did not differ between the non-LC and the CON.<br>cfPWV was similar between the LC, the non-LC, and CON groups, p = 0.247. Raw cfPWV data is available as figures only.	6
Nandadeva et al. [107], 2023	Observa-tional cross-sectional	FMD, Logiq P5 ultrasound (GE, Milwaukee, WI)<br>cfPWV, SphygmoCor (AtCor XCEL 1.3 software, Sydney, Australia)	In very-late recovery:<br>FMD did not differ between the LC: 4.38% and the non-LC group: 5.98%, p > 0.05 (raw FMD data in percentage available as figures only, parameters calculated based on mean change in absolute brachial artery diameter).<br>cfPWV was higher in the LC: 7.1 (1.2) m/s compared to the non-LC group: 6.0 (0.8) m/s, p = 0.015.<br>cfPWV and FMD were not correlated with a total symptom burden, p > 0.05.	7
Oikonomou et al. [28], 2022	Observational cohort	FMD, Vivid Ultrasound (GE, Milwaukee, Wisconsin, USA)	FMD was impaired: in acute COV: 1.65 (2.31)% vs. CON: 6.51 (2.91)%, p < 0.001; in early recovery: 4.23 (2.02)% vs. CON: 6.48 (3.08)%, p < 0.001; in late recovery: 5.24 (1.62)% vs. CON: 6.48% (3.08), p = 0.01.<br>ICU-treated subjects presented impaired FMD compared to those treated in the medical ward: in acute COV: 0.48 (1.01)% vs. CON: 2.33 (2.57)%, p = 0.001; in early recovery: 2.29	9

			(0.86)% vs. CON: 4.63 (1.96)%, p = 0.001; in late recovery: 3.18 (0.69)% vs. CON 5.67 (1.41)%, p < 0.001. In late recovery, there were no differences in FMD according to the presence or absence of the LC syndrome: 4.98 (1.90)% vs. 5.02 (1.15)%, p = 0.42.	
Oikonomou et al. [99], 2023	Observational case–control	cf-PWV, SphygmoCor device (AtCor Medical)	Post-COV individuals presented with impaired cfPWV compared to CON in early recovery: post-COV 12.1 (3.2) m/s vs. CON 9.6 (1.9) m/s, p < 0.001; and in late recovery: post-COVID 11.7 (2.7) m/s vs. CON 9.6 (1.9) m/s, p < 0.001. There were no differences between post-COV participants with and without LC syndrome (p value not given).	9
Oliveira et al. [90], 2021	Observational cohort	FMD, Sonosite turbo M (Fujifilm, Bothell, WA, USA)	FMD was lower in acute COV than CON, p < 0.01. Relative FMD ≤ 3.43% (sensitivity 85%, specificity 84%, AUC 0.922) predicted mortality and more extended hospital stay. Raw FMD data is available as figures only.	9
Province et al. [91], 2022	Observational cross-sectional	FMD, Logiq eR7 ultrasound and L4-12T-RS transducer (GE Medical Systems)	FMD was lower among post-COV groups compared to the healthy CON: 9.30 (2.73); in subacute COV (M1): 3.06 (1.39)%, p < 0.001, Hedge's g = 2.816) in early recovery (M2): 4.10 (2.03)%, p < 0.001, Hedge's g = 2.349), and (M3): 3.91 (2.64)%, p < 0.001, Hedge's g = 2.434) in mid-term recovery (M4): 4.40 (1.90)%, p < 0.001, Hedge's g = 2.215), and (M6): 6.60 (2.07)%, p = 0.001, Hedge's g = 1.223)	7
Ratchford et al. [89], 2021	Observational cross-sectional	FMD, Logiq eR7 ultrasound, and L4-12T-RS Transducer (GE Medical Systems, Milwaukee, WI) cfPWV, unspecified device	In subacute COV: FMD was 6% lower in the post-COV: 2.71 (1.21)% vs. the healthy CON: 8.81 (2.96)%, p < 0.01. cfPWV was 0.75 m/s higher in the post-COV: 5.83 (0.62) m/s vs. the healthy CON: 5.17 (0.66) m/s, p < 0.01.	7
Riou et al. [103], 2021	Observational cross-sectional	FMD, unspecified device	In mid-term recovery, FMD was lower in the post-COV: 8.1 (7.2-8.9) vs. CON: 10.3 (9.1-11.7), p = 0.002, with 44% of the hospitalized post-COV presented with a reduced FMD (< 8%). Impaired FMD was not associated with severe or critical acute COV.	6
Schnaubelt et al. [33], 2021	Observational case-control	cfPWV, BOSO ABI Systems 100 PWV® (Bosch & Sohn GmbH, Jungingen, Germany)	In acute COV, cfPWV was higher in the COV group: 14.3 (13.4–16.0) m/s vs. CON: 11.0 (9.5–14.6) m/s, p = 0.007. cfPWV was higher among COV fatalities vs. survivors (p = 0.056). In COV survivors, cfPWV correlated with the duration of hospital stay (r = 0.689, p = 0.019).	9
Skow et al. [96], 2022	Randomized controlled trial	FMD, Logiq P5 (GE Healthcare, Illinois, USA) cfPWV, SphygmoCor XCEL 1.3 (Atcor Medical, Sydney, Australia)	In early recovery, both FMD and cfPWV did not differ between the post-COV and vaccinated healthy CON: for FMD, the post-COV: 6.1 (2.3)% vs. CON: 5.9 (2.8)%, p = 0.544 for cfPWV, the post-COV: 5.7 (0.8) m/s vs. CON: 5.9 (0.6) m/s, p = 0.367	7

Tudoran et al. [100], 2023	Observa-tional cohort	cfPWV, SphygmoCor device (AtCor Medical, Sydney, NSW, Australia)	In early recovery, cfPWV was higher in the post-COV with MS: 10 (9–12) m/s vs. the post-COV without MS: 10 (9–11) m/s, p = 0.015, and between both post-COV groups and CON: 7 (6-7), p < 0.001.<br>Altered cfPWV correlated, among others, with lung injury (r = 0.63, p < 0.0001), time elapsed since COV diagnosis (r = -0.66, p < 0.0001), PCFS level (r = 0.56, p < 0.0001) and number of MS factors (r = 0.41, p < 0.0001).	8
van der Sluijs et al. [104], 2023	Observational cross-sectional	cfPWV, ARTSENS Plus (Healthcare Technology Innovation Center, Indian Institute of Technology Madras, Chennai, India)	In mid-term recovery, cfPWV differed between LC subgroups of 31 participants: 6.2 (5.2-6.8) m/s vs. CON: 7.3 (6.2-8.4) m/s, p = 0.04.<br>However, cfPWV did not differ between 97 post-COV (with or without LC) compared to CON, p = 0.37. Raw cfPWV data is available as figures only.	8
Vidya et al. [106], 2023	Observational cross-sectional	cfPWV, BOSO ABI Systems 100 PWV® (Bosch & Sohn GmbH, Jungingen, Germany)	In mid-term recovery, cfPWV was higher among the post-COV with hypertension (group II): 12.07 (2.37) m/s, p 0.001, and the post-COV with obesity (group III): 10.25 (2.54) m/s, p < 0.001, when compared to CON: 9.80 (2.10) m/s and 8.20 (1.22), respectively. However, cfPWV did not differ between the post-COV with DM (group I): 8.29 (1.52) and CON: 7.85 (1.11), p = 0.104.<br>cfPWV was the highest in the post-COV with hypertension, followed by the post-COV with obesity, and the post-COV with DM (p values not given).	6
Zanoli et al. [35] (Study 1), 2022	Observational cross-sectional	cfPWV, SphygmoCor system (AtCorMedical, Sydney, Australia)	cfPWV was higher in the post-COV in mid-term recovery: 9.0 (2.4) m/s vs. CON: 7.9 (1.5) m/s, p = 0.001, but not in very late recovery: 8.2 (1.3) m/s, p = 0.78.<br>The cfPWV was negatively associated with the interval from acute disease to measurement, p = 0.045. The higher the number of persistent symptoms during the study, the higher the cfPWV, p = 0.001. Higher levels of sensitivity C-reactive protein at the acute COV was associated with higher cfPWV in F/U, p = 0.04.	7

ASCVD: atherosclerotic cardiovascular disease; AUC: area under the curve; BMI: body mass index; cfPWV: carotid-femoral pulse wave velocity; CI: confidence interval; CON: control group; COV: COVID-19; DM: diabetes; FMD: brachial flow-mediated dilation, LC: group with long-COVID-19 syndrome; MS: metabolic syndrome; non-LC: group without long-COVID-19 syndrome; NOS: Newcastle–Ottawa Scale; OR: odds ratio; PCFS: post-COVID-19 functional scale

Supplementary Table S5. Study design and main findings in studies assessing changes in selected parameters during follow-up.

Study	Design	Assessment	Main findings	Quality (NOS)
Belcaro et al. [111], 2022	Prospective randomized controlled	FMD, Siemens X300 (Siemens Switzerland AG, Zurich, Switzerland)	In the non-Pycnogenol® group, FMD was low but improved from 7.2 (1.0)% to 8.0 (0.9)% during early recovery and to 8.8 (1.4)% during the mid-term recovery.	9

Gounaridi et al. [98], 2023	Prospective randomized controlled	FMD, Vivid Ultrasound (GE, Milwaukee, WI, USA)<br>cfPWV, SphygmoCor device (AtCor Medical, Sydney, NSW, Australia)	In the non-CR group, FMD improved from 5.9 (2.2)% in early recovery to 6.6 (1.8)% in mid-term recovery, $p = 0.009$.<br>However, cfPWV was not: 8.9 (1.8) m/s vs. 8.8 (1.9) m/s, $p = 0.74$.	9
Lambadiari et al. [11], 2021<br>Ikonomidis et al. [12], 2022	Observational case-control	FMD, Vivid E95 ultrasound (GE Medical Systems, Horten, Norway)<br>cfPWV, Complior system (Alam Medical, Vincennes, France)	FMD and cfPWV values were similar in mid-term and very late recovery.<br>FMD: 5.86 (2.82)% vs. 6.49 (2.25)%, $p = 0.198$.<br>cfPWV: 12.09 (2.50) m/s vs. 11.19 (2.53) m/s, $p = 0.776$.	8 and 8
Oikonomou et al. [28], 2022	Observational cohort	FMD, Vivid ultrasound (GE, Milwaukee, Wisconsin, US)	FMD improved during 6-month F/U, from the acute phase: 1.75 (2.19)% to the early recovery: 4.23 (2.02)%, and the late recovery period: 5.24 (1.62%), $p = 0.001$.	9
Oikonomou et al. [99], 2023	Observational case–control	cfPWV, SphygmoCor device (AtCor Medical)	cfPWV improved during 6-month F/U, from the early recovery: 12.1 (3.2) m/s to the late recovery period: 11.7 (2.7) m/s, $p = 0.11$.	9
Peng et al. [102], 2024	Interventional, pre-post	FMD, UNEXEF38G ultrasound (UNEX, Nagoya, Japan)<br>cfPWV, BOSO ABI Systems 100 (Bosch & Sohn, Germany)	In early recovery, but not in mid-term recovery, FMD decreased compared to pre-COV.<br>Pre-COV: 12.65 (10.30–15.38)% vs. early recovery: 10.80 (9.68–11.55)%, $p = 0.044$.<br>Pre-COV: 11.00 (10.45–12.95) % vs. mid-term recovery: 11.10 (10.30–11.35)%, $p = 0.290$.<br>There was no increase in cfPWV between the pre-and post-COV periods.<br>Pre-COV: 5.97 (0.66) m/s vs. early-recovery: 6.33 (1.00) m/s, $p = 0.172$.<br>Pre-COV: 5.74 (0.74) m/s vs. mid-term recovery: 6.27 (1.03) m/s, $p = 0.067$.	6
Podrug et al. [101], 2023	Interventional, pre-post	cfPWV, Sphygmocor CvMS, and SphygmoCor Xcel device (Atcor Medical, Sydney, Australia)	An average increase of 0.19 m/s (95% CI -0.04 to 0.41) in cfPWV was noted from the pre-COV period: 6.3 (0.7) m/s to the early recovery period: 6.5 (1.0) m/s, $p = 0.052$.<br>Age ($p = 0.005$) and time since acute-COV ($p = 0.030$) were positively associated with the cfPWV change.	6
Province et al. [91], 2022	Observational cross-sectional	FMD, Logiq eR7 ultrasound and L4-12T-RS transducer (GE Medical Systems)	FMD did not increase from subacute COV (M1): 3.06 (1.39)% to the early recovery period: (M2) 4.10 (2.03)%, $p = 0.453$, Hedge's $g = -0.509$, and (M3) 3.91 (2.64)%, $p = 0.850$, Hedge's $g = -0.417$, and to the mid-term recovery period in 4-th month (M4) 4.40 (1.90)%, $p = 0.228$, Hedge's $g = -0.656$.<br>However, FMD significantly improved from subacute COV (M1) to mid-term recovery in the sixth month (M6): 6.60 (2.07)%, $p < 0.001$, Hedge's $g = -1.737$.	7

Saloň et al. [14], 2023	Observational cohort	cfPWV, VICORDER® device (SMT medical GmbH & Co. KG, Würzburg, Germany)	The trend of increasing cfPWV from subacute COV: 11 (3) m/s to early recovery: 12 (3) m/s, p = 0.095, was recorded.	6
Szeghy et al. [110], 2022	Observational cross-sectional	cfPWV, SphygmoCor CPv (AtCor Medical, Sydney, Australia)	The cfPWV decreased from subacute COV: 5.70 (0.73) m/s to mid-term recovery: 4.88 (0.65) m/s, p < 0.05.	7
Teixeira DO Amaral et al. [112], 2022	Prospective randomized	cfPWV, Complior Analyze™PWV and Central Pressure Analysis™ (AlamMedical, Saint-Quentin-Fallavier, France)	In the non-CR group, cfPWV was not improved during F/U, p = 0.043. The raw cfPWV data of the non-CR group are available as figures only.	7
Zanoli et al. [35]; (Study 2), 2022	Observational cohort	cfPWV, SphygmoCor system (AtCorMedical, Sydney, Australia)	During F/U, cfPWV was improved by 9% from mid-term recovery: 8.8 (2.0) m/s to very-late recovery: 8.0 (1.6) m/s, p = 0.01. At the end of F/U, cfPWV remained 6% higher in the post-COV than CON, p = 0.04.	8

cfPWV: carotid-femoral pulse wave velocity; CON: control group; COV: COVID-19; FMD: brachial flow-mediated dilation, F/U: follow up; non-CR group: patients non participated in cardiac rehabilitation; non-Pycnogenol® group: patients not receiving the supplement; NOS: Newcastle–Ottawa Scale
